# Foxo in T Cells Regulates Thermogenic Program through Ccr4/Ccl22 Axis

**DOI:** 10.1016/j.isci.2019.11.006

**Published:** 2019-11-07

**Authors:** Tetsuhiro Kikuchi, Jun Nakae, Yoshinaga Kawano, Nobuyuki Watanabe, Masafumi Onodera, Hiroshi Itoh

**Affiliations:** 1 Navigation Medicine of Kidney and Metabolism, Division of Endocrinology, Metabolism, and Nephrology, Department of Internal Medicine, Keio University School of Medicine, Tokyo 160-8582, Japan; 2 Department of Physiology, International University of Health and Welfare School of Medicine, Narita 286-8686, Japan; 3 Department of Human Genetics, National Center for Child Health and Development, Tokyo 157-8535, Japan

**Keywords:** Molecular Mechanism of Behavior, Immunology, Immune Response, Diabetology

## Abstract

Crosstalk between immunity and the thermogenic program has provided insight into metabolic energy regulation. Here, we generated thermogenic program-accelerating mice (*T-QKO*), in which Foxo1 is knockout and Foxo3 is hetero-knockout in CD4^+^ T cells. *T-QKO* exhibit lean phenotype under HFD due to increased energy expenditure. Cold exposure significantly increased expression of the thermogenic genes (*Ppargc1a* and *Ucp1*), Th2 cytokines (*Il4* and *Il13*), and Th2 marker gene (*Gata3*) in subcutaneous adipose tissue (SC) of *T-QKO*. Furthermore, *Ccr4* expression was significantly increased in Th2 cells of *T-QKO*, and cold exposure induced *Ccl22* expression in SC, leading to increased accumulation of Th2 cell population in SC of *T-QKO*. These data reveal a mechanism by which cold exposure induces selective recruitment of Th2 cells into SC, leading to regulation of energy expenditure by generating beige adipocyte and suggest that inhibition of Foxo in T cells may support a strategy to prevent and treat obesity.

## Highlights

• *T-QKO* increases *Gata3* and *Ccr4* expression in CD4^+^ T cells
• Cold exposure increases *Ccl22* expression in subcutaneous adipose tissue
• Cold exposure increases SC-specific recruitment of Th2 cells in *T-QKO*
• Recruited Th2 cells secrete IL-4 and IL-13 and increase beiging of adipocytes

## Graphical Abstract

**Figure:**
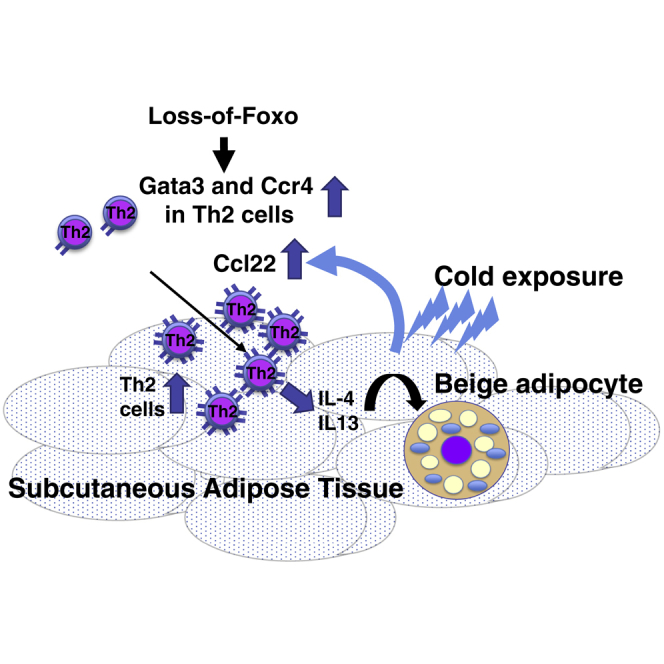

## Introduction

Obesity is an important disorder as a cause of metabolic syndrome, which represents insulin resistance, leading to type 2 diabetes, hypertension, hyperlipidemia, chronic renal disease, coronary artery disorder, and cerebrovascular disease (NCD Risk Factor Collaboration, 2016). Metabolic syndrome also causes chronic inflammation in adipose tissue by recruiting pro-inflammatory immune cells, including pro-inflammatory M1 macrophages, Th1/Th17 CD4^+^ T cells, and CD8^+^ T cells (Hotamisligil, 2006) (Hotamisligil, 2017).

Obesity results from a positive energy balance between energy intake, which is determined by food intake and/or energy absorption, and energy expenditure (Rosen and Spiegelman, 2006). Adaptive thermogenesis, which is defined as heat production in response to cold exposure or overfeeding, protecting the organism from cold or regulating energy balance after changes in diet, is important physiologically as one of the determinants of energy expenditure. Brown adipose tissue (BAT) and skeletal muscle are the two major organs involved in adaptive thermogenesis (Cannon et al., 1998). Rodents have prominent brown fat depots, whereas humans and other larger mammals do not; however, brown adipocytes may be dispersed among white adipose tissues (WAT) (Rosen and Spiegelman, 2006). Recent studies demonstrate that mammals have at least two types of thermogenic adipocytes, the classical brown adipocytes and inducible, termed beige adipocytes (Wu et al., 2012). Beige adipocytes emerge postnatally from WAT and are highly induced by various environmental stimuli, including chronic cold exposure, exercise, treatment with β3-agonist, and peroxisome proliferator-activated receptor-γ (PPARγ) activity (Kajimura et al., 2015).

Recent findings have shown that the crosstalk of brown and beige adipocytes with immune cells is important for thermogenic activation. The pro-inflammatory cytokines secreted by the infiltrating M1 macrophages of obese WAT might interfere with generation of beige adipocytes (Chiang et al., 2009) (Chung et al., 2017). In contrast, non-inflammatory, alternatively activated M2 macrophages support the thermogenic activity and sympathetic tone of BAT and beige adipose tissue (Nguyen et al., 2011) (Qiu et al., 2014). Various immune cell types, including macrophages, eosinophils, and group 2 innate lymphoid cells (ILC2s), act inside adipose tissues to govern the thermogenic activation and recruitment of brown and beige adipose tissues (Villarroya et al., 2018). Using loss-of-function approaches (Stat6-deficient or IL4Rα-deficient mice), researchers showed that type 2 cytokine signaling is necessary for the proper development of the thermogenic response in BAT (Nguyen et al., 2011). Furthermore, type 2 signaling is important for browning of WAT, which is triggered by cold exposure and by multiple other signals (Fabbiano et al., 2016, Suarez-Zamorano et al., 2015). However, little is known about the molecular mechanism of how chronic cold exposure induces type2 immune signaling in adipose tissues.

Foxo family members, including Foxo1, Foxo3, Foxo4, and Foxo6, are phosphorylated, subsequently exported to the cytoplasm; they are inhibited by insulin/IGF1 in a PI3 kinase-dependent manner and activated by nuclear localization due to oxidative stress (Nakae et al., 2008). These transcription factors are central to the integration of growth factor signaling, oxidative stress, and immunological inflammation and provide a connection between physical well-being and the form and magnitude of an immune response. There is a role for Foxo transcription factors in almost every aspect of T cell biology (Hedrick et al., 2012). However, whether or not Foxo transcription factors in T cells regulate glucose and energy metabolism is unknown.

In the present study, we demonstrated that high-fat diet (HFD) activates Foxo1 in CD4^+^ T cells and CD4^+^ T-cell-specific *Foxo1* knockout, and *Foxo3* hetero-knockout mice (*T-QuarterKO* [*T-QKO*]) exhibit an anti-obese phenotype due to increased energy expenditure under HFD. *T-QKO* show increased expression of the type 2 cytokines, IL-4 and IL-13, in adipose tissues, due to increased expression of *Gata3* and *Ccr4* in Th2 cells, and selective recruitment of Th2 cells to adipose tissues due to cold-induced expression of *Ccl22* in adipose tissues. These data indicate that Foxo transcription factors in CD4^+^ T cells regulate selective homing of Th2 cells to adipose tissues and beiging of white adipocytes, implicating the crosstalk between immunity and metabolism.

## Results

### High-Fat Diet Activates Foxo1 in CD4^+^ T Cells in Adipose Tissue

Obesity is characterized by a low-grade inflammatory state in adipose tissue (Lumeng et al., 2007) (Mathis, 2013). Adipose tissue macrophages (ATMs) infiltrate adipose tissue and secrete inflammatory cytokines, inhibiting the insulin signal in insulin-sensitive tissues, including liver, adipose tissue, and muscle (Hotamisligil, 2006). Evidences has accumulated that the adaptive immune system, including the infiltration of both T helper and cytotoxic cells into adipose tissue, also participates in the inflammatory response to obesity (Nishimura et al., 2009) (Yang et al., 2010).

Foxo family members, especially Foxo1 and Foxo3, have an important physiological role in CD4^+^ T cells, as indicated by the fact that double-knockout mice of both Foxo1 and 3 are lethal at the age of 8–12 weeks due to a fatal inflammatory disorder (Ouyang et al., 2010). Foxo family transcription factors are phosphorylated and inactivated in a PI3-kinase-dependent manner. Therefore, at fed state under normal chow diet (NCD), Foxo1 is usually localized in the cytoplasm and inactivated in several insulin-responsive tissues, including liver, adipose tissue, adipose tissue macrophages, and pancreatic β-cells (Nakae et al., 2008). However, under long-term HFD, nuclear localization of Foxo1 in adipose tissue macrophages is significantly increased, probably due to increased oxidative stress (Kawano et al., 2012). Therefore, excessive calorie intake sometimes changes environmental nutritional circumstances in adipose tissue and might change Foxo activity.

**Figure 1:**
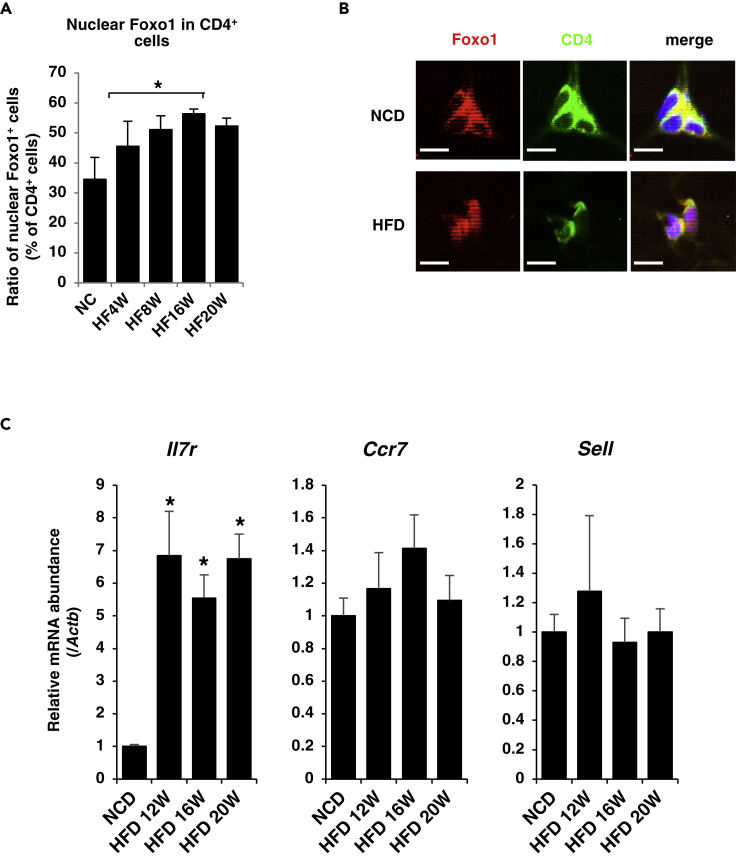
HFD Activates Foxo1 in CD4^+^ T Cells of Epididymal Adipose Tissues (A) The percentage of nuclear Foxo1 in CD4^+^ T cells of epididymal adipose tissues from age-matched C57Bl6/J mice fed HFD (N = 4). *p < 0.05 by one-way ANOVA. (B) Representative immunofluorescence of Foxo1 and CD4 in epididymal fat from age-matched control mice fed with an NCD and 16-week HFD. Scale bar, 10μm. (C) Normalized gene expression of *Il7r*, *Ccr7*, and *Sell* in CD4^+^ T cells sorted from epididymal adipose tissues of C57Bl6/J mice fed with HFD for the indicated duration (n = 8–10). Values were normalized to β-actin expression and represented as the ratio to value of NCD. *p < 0.05 by one-way ANOVA.

To investigate the effects of HFD on Foxo1 activity in CD4^+^ T cells in adipose tissue, we examined intracellular localization of Foxo1 in CD4^+^ T cells of adipose tissues from age-matched C57Bl6/J mice fed with HFD for 20 weeks. HFD increases nuclear localization of Foxo1 in CD4^+^ T cells of epididymal fat significantly (Figures 1A and 1B). Furthermore, HFD significantly increases *Il7r* compared with NCD and tends to increase *Ccr7*, which are target genes of Foxo1 in T cells (Ouyang et al., 2009) (Luo and Li, 2018), in CD4^+^ T cells (Figure 1C). These data suggest that HFD activates Foxo1 in CD4^+^ T cells in adipose tissue.

### *T-QKO* Mice Exhibit Anti-obese Phenotype under HFD

CD4^+^ T-cell-specific *Foxo1* and *Foxo3* double-knockout mice (*T-DKO*) are lethal from 8 weeks of age due to immunological disturbance. However, single knockout of *Foxo1* or *Foxo3* in CD4^+^ T cells revealed no lethal phenotype, suggesting that both Foxo1 and Foxo3 in CD4^+^ T cells have a redundant function with each other and that both Foxo1 and Foxo3 are indispensable for the physiological function of CD4^+^ T cells (Ouyang et al., 2010). However, the physiological roles of Foxo1 and Foxo3 in CD4^+^ T cells with respect to glucose and energy metabolism have not been reported. Therefore, in order to investigate the pathophysiological roles of Foxo1 and Foxo3 in CD4^+^ T cells and their potential effects on glucose and energy metabolism, we generated CD4^+^ T-cell-specific Foxo1 single-knockout (*T-Foxo1KO*) and Foxo3 single-knockout (*T-Foxo3KO*) mice (Figure S1).

Foxo1 expression level in CD4^+^ T cells isolated from *T-Foxo1KO* mice was significantly decreased by 90% compared with control mice (Figure S2A). Body weight, glucose tolerance, and insulin tolerance tests revealed no significant differences between control and *T-Foxo1KO* mice under NCD (data not shown). There were also no significant differences in body weight, glucose tolerance, or insulin sensitivity between control and *T-Foxo1KO* mice under HFD (Figures S2B–S2D). In addition, *Foxo3* expression in CD4^+^ T cells isolated from *T-Foxo3KO* mice was decreased by 70% compared with controls (Figure S2E). Body weight, glucose tolerance, and insulin tolerance tests of *T-Foxo3KO* revealed no significant differences between control and *T-Foxo3KO*, whether under NCD (data not shown) or HFD (Figures S2F–S2H). These data suggest that Foxo1 and Foxo3 in CD4^+^ T cells are each individually dispensable for the regulation of glucose and energy metabolism.

**Figure 2:**
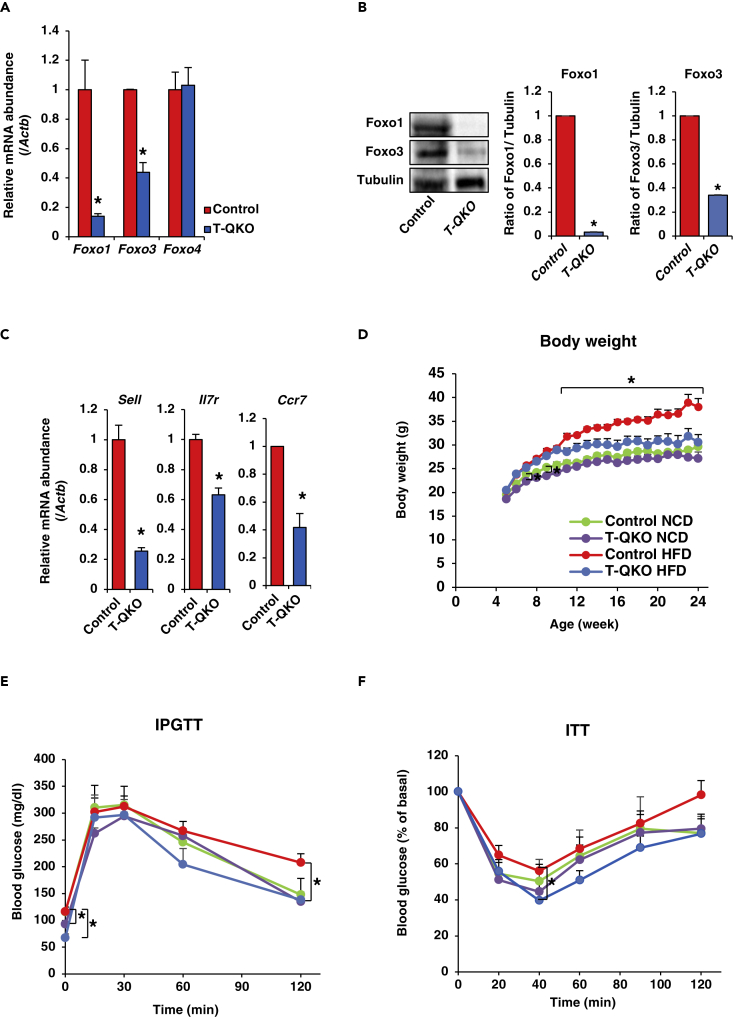
*T-QuarterKO* (*T-QKO*) Exhibit Anti-Obese Phenotype under HFD (A) Expression of *Foxo1*, *Foxo3*, and *Foxo4* in CD4^+^ T cells sorted from spleen of control and *T-QKO* (n = 4). Data are normalized to β-actin expression. Data are means ± SEM. *p < 0.05 by one-way ANOVA. (B) Foxo1 and Foxo3 protein expression in CD4^+^ T cells sorted from spleen of control and *T-QKO*. The left panel shows the representative western blotting. The right panel indicates normalization of the ratio of density of Foxo1 or Foxo3 to tubulin in sorted CD4^+^ T cells from control and *T-QKO* (n = 4). Data are the ratio to the density of control and represent means ± SEM. *p < 0.05 by one-way ANOVA. (C) Normalized gene expression of *Sell*, *Il7r*, *and Ccr7* in CD4^+^ T cells sorted from spleen of control and *T-QKO* (n = 4). Values were normalized to β-actin expression and represented as the ratio to value of NCD. *p < 0.05 by one-way ANOVA. (D) Body weight of control, *T-QKO* mice fed with NCD (n = 13), control, and *T-QKO* mice fed with HFD (n = 13). Data are means ± SEM. *p < 0.05 by two-way ANOVA with Fisher's test. (E) IPGTT of control, *T-QKO* mice fed with NCD at the age of 21 weeks (n = 5), control, and *T-QKO* mice fed with 16-week HFD at the age of 21 weeks (n = 7). Data are means ± SEM. *p < 0.05 by two-way ANOVA with Fisher's test. (F) ITT of control, *T-QKO* mice fed with NCD at the age of 21 weeks (n = 4), control, and *T-QKO* mice fed with 16-week HFD at the age of 21 weeks (n = 11). Data are means ± SEM. *p < 0.05 by two-way ANOVA with Fisher's test.

Next, we generated CD4^+^ T-cell-specific *Foxo1* knockout *Foxo3* hetero-knockout mice, in which the gene dosage of both *Foxo1* and *Foxo3* alleles in CD4^+^ T cells was reduced to 25% of control. Therefore, we named these mice *T-QuarterKO* (Figure S1). Real-time PCR demonstrated that *Foxo1* expression was reduced by 90% and *Foxo3* expression was reduced by approximately 45% in CD4^+^ T cells isolated from *T-QKO* (Figure 2A). Furthermore, western blotting demonstrated that CD4^+^ T cells sorted from spleen of *T-QKO* expressed around 3% of Foxo1 protein and around 30% of Foxo3 protein of control CD4^+^ T cells (Figure 2B). These data indicated that Foxo family members in CD4^+^ T cells from *T-QKO* are expressed at approximately 25% of the levels observed in control CD4^+^ T cells. Consistent with these data, expression levels of Foxo target genes in CD4^+^ T cells isolated from *T-QKO*, which include *Sell*, *Il7r*, and *Ccr7*, were significantly reduced compared with controls (Figure 2C).

In order to investigate the pathophysiological effects of Foxo family members in CD4^+^ T cells on glucose and energy metabolism, we examined body weight, glucose tolerance, and insulin tolerance. Under NCD, body weight, glucose tolerance, and insulin tolerance of *T-QKO* mice were similar to controls (Figures 2D–2F). In contrast, under HFD, *T-QKO* mice exhibited significantly reduced body weight compared with controls (Figure 2D). Food intake and triglyceride content in stool were similar between control and *T-QKO* mice (Figures S3A and S3B). Furthermore, histological analysis of colon and small intestine revealed no apparent differences between control and *T-QKO* mice. The depth of crypt and the number of goblet cells, which are hallmarks of intestinal inflammation (Kawano et al., 2016), in colon and small intestine of *T-QKO* were similar to control (Figures S3C and S3D). These findings indicate no cachexic nor malabsorptive phenotypes in *T-QKO* mice. Furthermore, fasting blood glucose, glucose tolerance, and insulin sensitivity of *T-QKO* mice were significantly improved compared with controls under HFD (Figures 2E and 2F). During the intraperitoneal glucose tolerance test, insulin secretion tended to be lower in *T-QKO* mice than in controls but did not differ significantly (Figure S3E).

CD4^+^ T-cell-specific *Foxo1* hetero-knockout *Foxo3* knockout mice (*T-reverseQKO*; *T-rQKO*) were also generated (Figure S1). Body weight, glucose tolerance, and insulin tolerance tests were similar between *T-rQKO* and control mice under NCD (Figures S4A–S4C), as well as under HFD (Figures S4D–S4F). These data indicate that 75% reduction of *Foxo* expression in CD4^+^ T cells improves glucose and energy metabolism deteriorated by HFD and that 50% of the *Foxo1* allele, but not the *Foxo3* allele, in CD4^+^ T cells is indispensable for the regulation of glucose and energy metabolism.

### *T-QKO* Mice Exhibit Reduced Chronic Inflammation in Adipose Tissue

**Figure 3:**
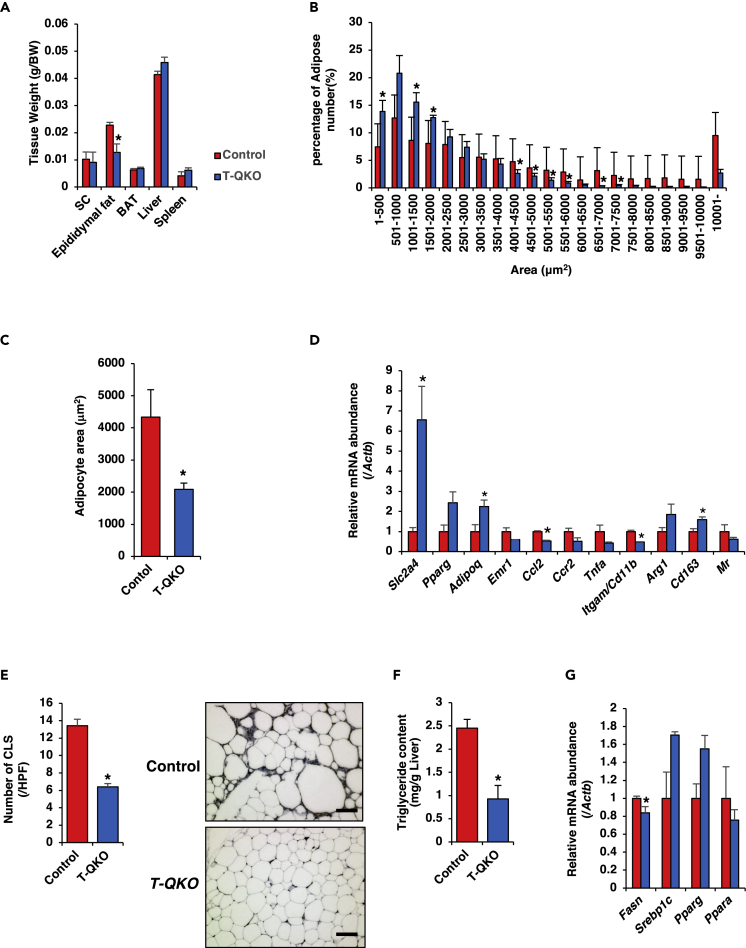
*T-QKO* Exhibited Decreased Chronic Inflammation in Adipose Tissue (A) Tissue weights of unilateral subcutaneous fat (SC), epididymal fat (Epi), whole BAT, liver and spleen from control (red bar) and *T-QKO* (blue bar) mice fed with a 15-week HFD. Data are the ratio of body weight and expressed as means ± SEM (n = 6). *p < 0.05 by one-way ANOVA. (B) Histogram of adipocyte size and number of epididymal fat from control (red bar) and *T-QKO* (blue bar) fed with a 15-week HFD (n = 6). Data represent percentage of total counted cells and means ± SEM. *p < 0.05 by one-way ANOVA. (C) Mean size of adipocytes of epididymal fat (n = 6). Data represent each adipocyte area (μm^2^) and means ± SEM. *p < 0.05 by one-way ANOVA. (D) Normalized gene expression of adipocyte-specific and immune-cell-related genes in epididymal fat in mice fed with a 20-week HFD (n = 6). Data are the ratio of control in each gene and means ± SEM. *p < 0.05 by one-way ANOVA. (E) The number of crown-like structures (CLSs) in epididymal fat of mice fed with a 20-week HFD (n = 6). Data represent the numbers of CLSs in 1 HPF (100 X) and means ± SEM. *p < 0.05 by one-way ANOVA. The right panels represent representative histological images with anti-CD68 antibody of epididymal adipose tissues (scale bar, 100 μm). (F) Triglyceride content in liver of control and *T-QKO* mice fed with a 20-week HFD (n = 6). Data are means ± SEM. *p < 0.05 by one-way ANOVA. (G) Normalized gene expression of fatty acid synthesis-related genes in liver in mice fed with a 20-week HFD (n = 6). Data are the ratio of control in each gene and means ± SEM. *p < 0.05 by one-way ANOVA.

*T-QKO* mice exhibit significantly reduced weight of epididymal fat (Figure 3A), as well as a significantly greater number of small adipocytes in epididymal fat (Figures 3B and 3C). HFD induces obesity and low-grade chronic inflammation in adipose tissue, particularly visceral adipose tissue (Lumeng et al., 2007) (Mathis, 2013). Therefore, we investigated the expression levels of adipose tissue-specific and pro-inflammatory genes. Real-time PCR demonstrated that expression levels of *Slc2a4* and *Adipoq*, which were correlated positively to insulin sensitivity (Kadowaki et al., 2006), in epididymal fat of *T-QKO* were significantly increased and expression levels of pro-inflammatory macrophage markers, including *Ccl2* and *Itgam*, were significantly decreased compared with control mice. In contrast, expression levels of marker genes of anti-inflammatory M2 macrophage, which include *Arg1* and *Cd163*, tended to be increased in *T-QKO* epididymal fat (Figure 3D). In contrast, expression levels of inflammatory genes in SC and BAT from *T-QKO* mice exhibited no significant changes compared with controls. Expression levels of marker genes of M2 macrophage in SC and BAT were not increased in *T-QKO* except *Cd163* in BAT (Figures S5A and S5B). Furthermore, immunohistochemistry of epididymal fat from *T-QKO* mice using anti-CD68 antibody exhibited significantly reduced numbers of crown-like structures (CLSs) versus controls (Figure 3E). However, the concentration of IL-1β, which was one of the proinflammatory cytokines, in peripheral blood of *T-QKO* was similar to control (Kawano et al., 2016) (Figure S5C). These data indicate that *T-QKO* mice exhibit reduced chronic inflammation in adipose tissue, especially in epididymal adipose tissue, under HFD and that this reduced chronic inflammation might be local. HFD also induces hepatic steatosis (Nanji, 2004). The hepatic triglyceride content of *T-QKO* mice was significantly reduced compared with controls (Figure 3F). The potential sources of fats contributing to fatty liver include dietary fatty acids, fatty acids newly made within the liver through *de novo* lipogenesis, and peripheral fats stored in white adipose tissue that flow into the liver (Postic and Girard, 2008). However, *Fasn* expression in liver of *T-QKO* was only reduced to 20% of control liver (Figure 3G). These data indicate that deletion of *Foxo* transcription factors in CD4^+^ T cells may improve hepatic steatosis by decreasing fats stored in adipose tissues, not by decreasing hepatic *de novo* lipogenesis.

### Intestinal Environment Dose Not Contribute to Phenotype in *T-QKO* Mice

The gut microbiome plays important roles in the regulation of glucose and energy homeostasis, and an HFD induces an increase in the proportion of Firmicutes, which have an increased capacity to harvest energy from the diet, to Bacteroides in the gut (Ley et al., 2005, Turnbaugh et al., 2006). Furthermore, intestinal adaptive immunity, including CD4^+^ T cells, affects metabolic regulation in obesity (Winer et al., 2016). Therefore, to investigate the effects of the loss-of-*Foxo* family members on the gut microbiome, first we analyzed the gut microbiome at phylum level. Usually, an HFD induces an increase in the ratio of Firmicutes to Bacteroides, controls intestinal permeability, and supports metabolic endotoxemia, leading to macrophage infiltration of adipose tissue (Cani et al., 2008, Everard and Cani, 2013). Analysis of cecum flora from *T-QKO* fed an HFD revealed a significant reduction of Bacteroides (Figure S5C). These data indicate that changes of gut microbiota do not seem to contribute to reduced inflammation in adipose tissues of *T-QKO*.

It has also been reported that eating an HFD leads to chronic inflammation that presents as infiltration by pro-inflammatory macrophages, which in turn leads to insulin resistance in peripheral insulin-responsive tissues, including adipose tissue and liver (Kawano et al., 2016). Therefore, to investigate whether improved glucose and energy metabolism in *T-QKO* mice were caused by reduced intestinal inflammation, we analyzed the expression of intestinal inflammatory genes. Analysis of gene expression in the colon and small intestine of *T-QKO* mice revealed increased expression of *Tnfa* and *Ccl2*, which are markers of pro-inflammation (Figures S5D and S5E). These data indicate that improved glucose tolerance and insulin sensitivity in *T-QKO* mice were not caused by decreased intestinal pro-inflammation.

### *T-QKO* Mice Exhibit Increased Energy Expenditure

**Figure 4:**
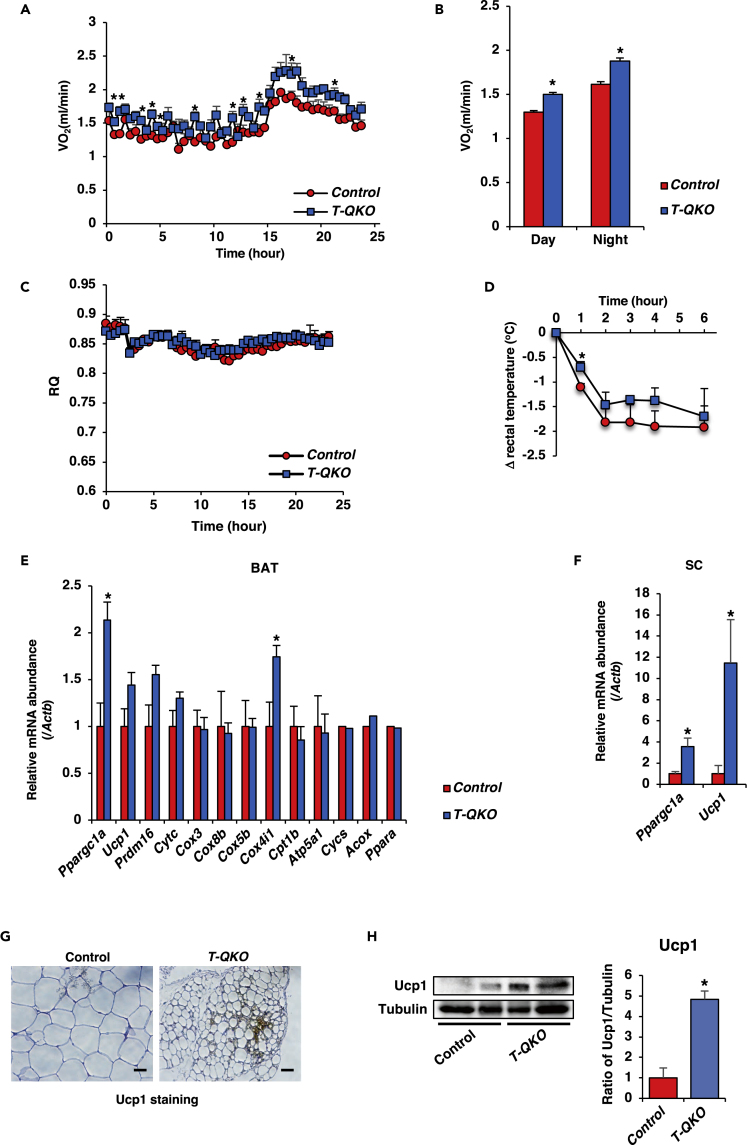
*T-QKO* Exhibit Increased Energy Expenditure and Activation of Thermogenic Program (A) The oxygen consumption (mL/min) of control (red circle) and *T-QKO* (blue square) fed with a 10-week HFD (n = 4). Data are means ± SEM. *p < 0.05 by two-way ANOVA with Fisher's test. (B) Means ± SEM of the oxygen consumption during day and night time. *p < 0.05 by one-way ANOVA. (C) Respiratory quotient (RQ) of control and T-QKO. Data are means ± SEM of four mice in each genotype. (D) Changes in rectal temperature of 16-week-old control (red circle) and *T-QKO* (blue square) fed with a 12-week HFD after cold exposure (n = 6). Data are means ± SEM. *p < 0.05 by two-way ANOVA with Fisher's test. (E) Normalized gene expression of thermogenic and mitochondrial genes in BAT from control and *T-QKO* fed with a 20-week HFD after 6-h cold exposure (4°C) (n = 6). Data are the ratio of control in each gene and means ± SEM. *p < 0.05 by one-way ANOVA. (F) Normalized gene expression of *Ppargc1a* and *Ucp1* in SC from control and *T-QKO* fed with a 20-week HFD after 6-h cold exposure (4°C) (n = 6). Data are the ratio of control in each gene and means ± SEM. *p < 0.05 by one-way ANOVA. (G) Representative images from UCP1 staining on section of SC from control and *T-QKO* fed with a 20-week HFD after 48-h cold exposure (scale bar, 20μm). (H) Representative image of western blotting of Ucp1 in SC from control and *T-QKO* fed with a 20-week HFD after 48-h cold exposure. The right panel indicates normalization of the ratio of density of Ucp1 to tubulin in SC from control and *T-QKO* (n = 4). Data are means ± SEM. *p < 0.05 by one-way ANOVA.

Body weight is known well to be regulated by energy intake, which includes food intake and absorption, and energy expenditure, which is determined by non-shivering thermogenesis and active locomotor activity (Rosen and Spiegelman, 2006). Therefore, we speculated that *T-QKO* mice would exhibit energy expenditure or increased locomotor activity. Although assessment of locomotor activity is out of our experimental design limit, we have not observed excessive and/or frustrated movement of *T-QKO* mice. Indirect calorimetry demonstrated that the oxygen consumption of *T-QKO* mice under HFD was significantly increased compared with controls but that the respiratory quotient was similar between them (Figures 4A–4C). Furthermore, exposing *T-QKO* mice at 4°C cold environment for 6 h revealed significant resistance to decline of rectal temperature compared with control mice (Figure 4D). These data indicate that *T-QKO* mice demonstrate increased energy expenditure versus control mice.

### *Foxo* Loss in CD4^+^ T Cells Activates a Thermogenic Program

Non-shivering thermogenesis occurs mainly in BAT (Rosen and Spiegelman, 2006). In addition, beige adipocytes, in which a thermogenic genetic program is installed in some white adipocytes of SC, reportedly play an important physiological role in non-shivering thermogenesis (Wu et al., 2012) (Wang and Seale, 2016). Expression levels of *Ucp1* and *Ppargc1a* in BAT and SC of *T-QKO* fed with NCD at room temperature were not different from control significantly (Figures S6A and S6B). Indeed, metabolic phenotypes, including body weight, of *T-QKO* were similar to control fed with NCD (Figures 2D–2F). Therefore, we focused on the analyses in mice fed with HFD.

To investigate the mechanism underlying how *T-QKO* mice exhibit increased energy expenditure, we analyzed gene expression in BAT and SC in mice that had been exposed to the cold for 6 h. Real-time PCR demonstrated that, compared with control mice, expression levels of *Ppargc1a* and *Cox4i1*, an isoform of the terminal oxidase in mitochondrial electron transport, were significantly increased in BAT from *T-QKO* mice, and *Ppargc1a* and *Ucp1* expression were significantly increased in SC from *T-QKO* mice (Figures 4E and 4F). Furthermore, immunohistochemistry demonstrated that cold exposure for 48 h significantly increased Ucp1-positive beige adipocytes in SC from *T-QKO* mice versus control mice (Figure 4G) and western blotting demonstrated that the expression levels of Ucp1 protein in SC from *T-QKO* was significantly increased compared with control after 48-h cold exposure (Figure 4H). These data indicate that reduced expression of Foxo family members in CD4^+^ T cells activates a thermogenic program in BAT and SC.

### *Foxo* Loss in CD4^+^ T Cells Increases *Gata3* and Th2 Cytokines in Adipose Tissues

**Figure 5:**
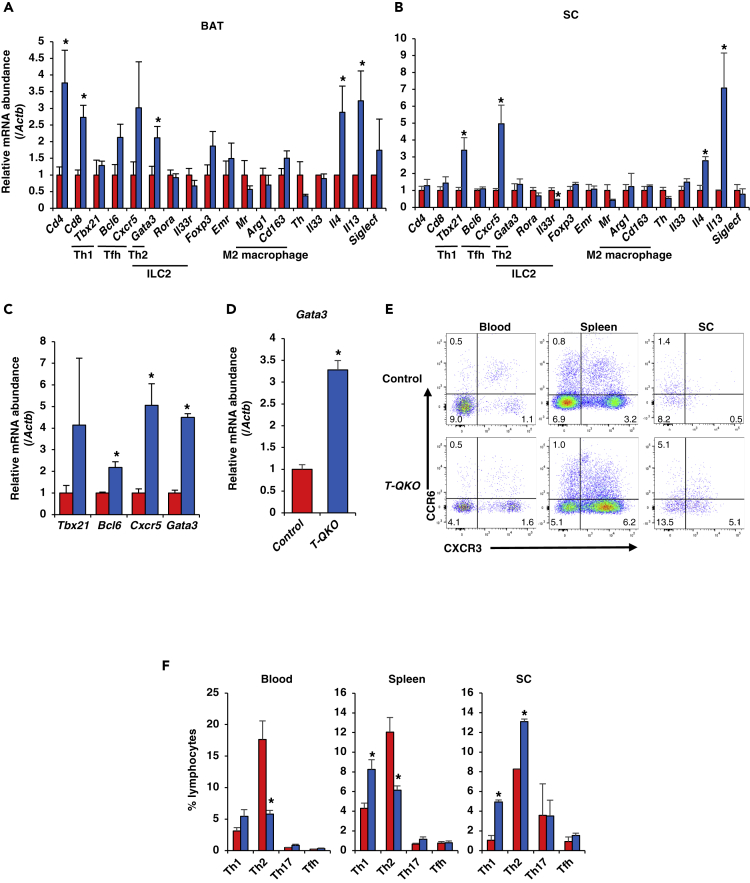
*T-QKO* Increase Th2 Cells in Subcutaneous Adipose Tissue upon Cold Exposure (A and B) Normalized gene expression of immune cell- and cytokine-related genes in BAT (A) and SC (B) from control and *T-QKO* fed with a 20-week HFD after 6-h cold exposure (4°C) (n = 6). Data are the ratio of control in each gene and means ± SEM. *p < 0.05 by one-way ANOVA. (C) Normalized gene expression of T-cell-related genes in CD4^+^ T cells sorted from SC of control and *T-QKO* fed with a 20-week HFD after 6-h cold exposure. Data are the ratio of control in each gene and means ± SEM. *p < 0.05 by one-way ANOVA. (D) Normalized gene expression of *Gata3* in CD4^+^ CCR6^−^ CXCR3^−^ Th2 cells FACS-sorted from spleen of control (red bar) and *T-QKO* (blue bar) mice at 4°C for 12 h (n = 4). Data are means ± SEM. *p < 0.05 by one-way ANOVA. (E) Surface CXCR3 and CCR6 expression of CD4^+^ lymphocytes. FACS analysis of CD4^+^ lymphocytes from peripheral blood (Blood), spleen, and subcutaneous adipose tissue (SC) of control and *T-QKO* fed with a 20-week HFD incubated at 4°C for 12 h (n = 4). (F) Bar graphs represent frequency of Th1, Th2, Th17, and Tfh cells. Data are the percentage of total lymphocytes and means ± SEM. *p < 0.05 by one-way ANOVA.

Immune cells are instrumental in the activation of brown adipocytes and the browning of white adipocytes (Chiang et al., 2009, Nguyen et al., 2011). Th2 cytokines, including IL4 and IL13, are key factors that activate brown adipocytes and enhance beige adipogenesis. Th2 cytokines are secreted mainly by eosinophils and ILC2s (Brestoff et al., 2015). CD4^+^ Th2 and T follicular helper (Tfh) cells also secrete IL4 or IL13. These types of CD4^+^ T cells have distinctive master transcription factors, namely Gata3 and Bcl6, respectively (Bao and Reinhardt, 2015, Walker and McKenzie, 2018). In order to investigate the mechanism by which the thermogenic program was increased in adipose tissues of *T-QKO* mice, expression levels of cytokines and transcription factors, which are related to type 2 immunity, were examined. Real-time PCR of BAT from *T-QKO* mice after 6 h of cold exposure demonstrated that *Il4* and *Il13* gene expression were significantly increased in *T-QKO* compared with control mice (Figure 5A). In addition, expression levels of *Gata3*, which is a master gene of Th2 cells, was significantly increased compared with controls. *Bcl6*, a master gene of Tfh cells, and *Cxcr5*, a surface marker of Tfh, had a non-significant tendency toward increase (Figure 5A). Recently, it has been suggested that Th2 cytokines increase alternatively activated macrophages, which synthesize and secrete norepinephrine, leading to the activation of brown adipocytes and the increased browning of white adipocytes (Nguyen et al., 2011) (Qiu et al., 2014). However, in the present study, expression levels of the M2 macrophage marker genes, *Mr*, *Arg1*, and *Cd163*, were similar in BAT from *T-QKO* and control mice. In addition, gene expression levels of *tyrosine hydroxylase* (*Th*, a rate-limiting enzyme of catecholamine synthesis) (Thomas and Palmiter, 1997), *Rora* and *Il33r* (ILC2 markers) (Spits et al., 2013), and *Siglecf* (an eosinophilic marker) (Johnson et al., 2015) were similar in BAT from *T-QKO* versus control mice (Figure 5A).

Furthermore, real-time PCR of SC from *T-QKO* after 6 h of cold exposure demonstrated that *Il4* and *Il13* expression were also significantly increased compared with controls. *Cxcr5* expression was also increased significantly in *T-QKO* mice compared with controls (Figure 5B). However, expression of *Il33r*, a receptor for IL-33 in ILC2s, was significantly reduced, and *Siglecf* and *Th* expression in SC were similar between *T-QKO* and control mice (Figure 5B). Finally, to investigate the effects of reduced gene expression of Foxo family genes in CD4^+^ T cells, real-time PCR was performed using CD4^+^ T cells sorted from SC by magnetic-activated cell sorting (MACS). Real-time PCR demonstrated that *Bcl6*, *Cxcr5*, and *Gata3* gene expression were significantly increased compared with controls (Figure 5C). Furthermore, *Gata3* expression in Th2 cell population (CD4^+^ CXCR3^−^ CCR6^−^) sorted from spleen of *T-QKO* was significantly increased compared with control (Figures S7A and 5D). These data confirm and indicate that reduced expression of *Foxo* family genes in CD4^+^ T cells increases *Gata3* expression, a master gene of Th2 cells, and Th2 immunity-related gene expression in adipose tissues.

### Cold Exposure Increases Th2 Cell Number in SC from *T-QKO* Mice

To investigate which kinds of immune cells are involved in activated thermogenesis in *T-QKO* mice, fluorescence-activated cell sorting (FACS) analyses were performed although without isotype control analysis (Figure S7A). CD4^+^ T cells in the spleen of *T-QKO* mice were significantly increased compared with controls; SC and BAT also tended to be increased, but not significantly so at room temperature (Figure S7B). In contrast, CD8^+^ T cells in spleen and SC from *T-QKO* were significantly reduced at room temperature (Figure S7C). Th1 cells were also significantly increased in the spleen of *T-QKO* mice compared with controls and also tended to be increased in SC and BAT, but not significantly so (Figure S7D). However, Th2 and Tfh cells in spleen, SC, and BAT were similar between control and *T-QKO* at room temperature (Figure S7D). These data indicate that the loss of *Foxo* in CD4^+^ T cells does not affect the numbers of Th2 or Tfh cells in SC and BAT at room temperature.

The thermogenic program is sometimes activated by several kinds of stimulus, including cold exposure. Therefore, FACS analysis was performed using samples from animals under incubation at 4°C for 12 h. CD4^+^ T cells in peripheral blood from *T-QKO* mice were significantly reduced compared with controls. In contrast, CD4^+^ T cells in SC from *T-QKO* mice tended to be increased compared with controls but not significantly different (Figures S8A and S8B). These data may indirectly indicate that the adipose tissue-specific homing of CD4^+^ T cells is increased in *T-QKO*. CD8^+^ T cells in spleen from *T-QKO* were significantly reduced; peripheral blood and SC also tended to be reduced (Figures S8A and S8C). Th1 cells were significantly increased in the spleen and SC of *T-QKO* mice compared with controls (Figures 5E and 5F). Th2 cells in peripheral blood and spleen from *T-QKO* were significantly reduced. In contrast, Th2 cells in SC from *T-QKO* mice were significantly increased compared with control mice (Figures 5E and 5F). These data indicate that cold exposure increases Th2 cell population in SC, but not in spleen and blood, from *T-QKO*.

### Adipose Tissue-Specific Homing of Th2 Cells Regulates the Thermogenic Program

**Figure 6:**
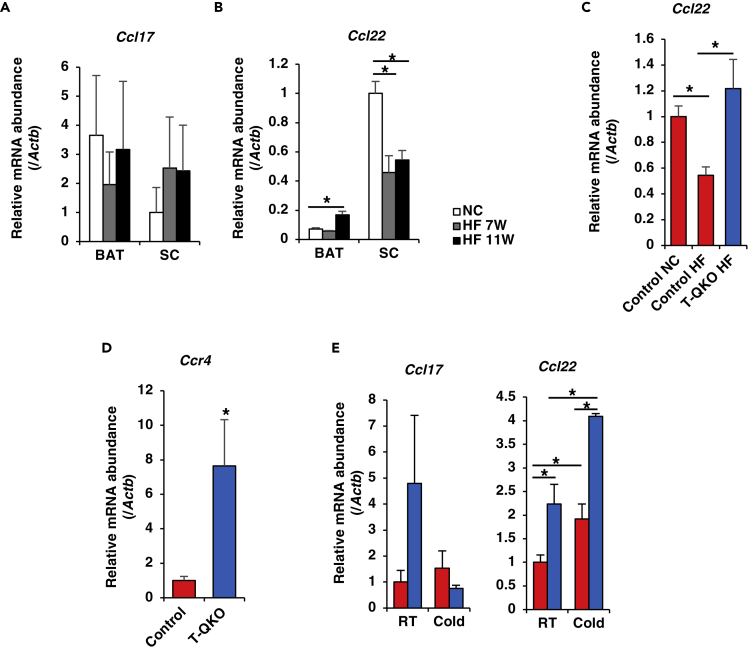
The Chemokine-Chemokine Receptor System Regulates Cold-induced Th2 Cell Accumulation and the Effects of HFD on Th2 Signaling-related Genes Expression in SC (A) Normalized gene expression of *Ccl17* in BAT and SC from control mice fed an HFD at room temperature (n = 5). (B) Normalized gene expression of *Ccl22* in BAT and SC from control fed an HFD at room temperature (n = 5). Data are means ± SEM. *p < 0.05 by one-way ANOVA. (C) Normalized gene expression of *Ccl22* in SC from control and *T-QKO* fed an HFD for 12 weeks at room temperature (n = 5). Data are means ± SEM. *p < 0.05 by one-way ANOVA. (D) Normalized gene expression of *Ccr4* in CD4^+^ CCR6^−^ CXCR3^−^ Th2 cells FACS-sorted from spleen of control (red bar) and *T-QKO* (blue bar) mice at 4°C for 12 h (n = 4). Data are means ± SEM. *p < 0.05 by one-way ANOVA. (E) Normalized gene expression of *Ccl17* and Ccl22 in SC from control and *T-QKO* mice at room temperature and 4°C for 12 h (n = 4). Data are means ± SEM. *p < 0.05 by one-way ANOVA.

Chemokines and chemokine receptors orchestrate cell migration and homing. Migration of immune cells is induced by chemoattractant receptors and their ligands, including chemokines. During inflammation, ligands for chemoattractant receptors are upregulated in tissue and vascular beds and provide directional cues for inflammatory T cells, on which the corresponding receptors are upregulated, to enter inflamed tissue from the blood (Islam and Luster, 2012, Luster et al., 2005). In order to investigate the mechanism by which cold exposure induces Th2 cell accumulation in SC, we focused on expression levels of chemokines and chemokine receptor related to Th2 cell. Among the various T cell subsets, Ccr4 is predominantly expressed in Th2 cells and is the receptor for two CC chemokine ligands (Ccls), which are Ccl17 and Ccl22 (Yoshie and Matsushima, 2015). Although HFD does not affect *Ccl17* expression in BAT and SC (Figure 6A), *Ccl22* expression level was significantly reduced in SC, but not in BAT (Figure 6B). Interestingly, *Ccl22* expression in SC from T-QKO fed an HFD was similar to control fed an NCD (Figure 6C). These data suggest that HFD suppresses *Ccl22* expression in SC.

*Ccr4* expression in CD4^+^ CXCR3^−^ CCR6^−^cell populations FACS-sorted from spleen of *T-QKO* mice at 4°C for 12 h was significantly increased compared with controls (Figure 6D). Furthermore, although *Ccl17* expression in SC from *T-QKO* mice was not significantly different from controls, *Ccl22* expression in SC from *T-QKO* mice was significantly increased compared with controls at both room temperature and 4°C for 12 h. Moreover, cold exposure induced *Ccl22* expression in SC from both animals (Figure 6E). These data indicate that *Foxo* loss increases *Ccr4* expression in Th2 cells and cold exposure induces *Ccl22* expression in SC, both leading to the increased accumulation of Th2 cells in SC from *T-QKO* mice.

## Discussion

In the present study, we demonstrated that HFD activates Foxo1 in CD4^+^ T cells in adipose tissues and that knockout of *Foxo1* hetero-knockout of *Foxo3* in CD4^+^ T cells causes increased homing of Th2 cells to SC due to induction of both *Ccr4* in Th2 cells and *Ccl22* expression in SC, resulting in increased beige adipocytes, in turn leading to increased whole-body energy expenditure and anti-obese phenotype in mice under HFD. In other words, Foxo family members in CD4^+^ T cells inhibit expression of *Gata3* and *Ccr4*, homing of Th2 cells to adipose tissues, leading to suppression of energy expenditure. These results describing the effect of a cold exposure on the homing of Th2 cells into adipose tissues suppose one of the mechanisms of browning of adipocytes at cold exposure.

Among Foxo-target genes, expression level of *Sell* was not changed significantly under HFD. It has been reported that *Il7r* and *Ccr7* were directly controlled by Foxo1 at transcriptional level. In contrast, *Sell* expression is regulated by Kruppel-like factor 2 (Klf2), which is induced by Foxo1 (Luo and Li, 2018). Therefore, the different transcriptional regulation may affect the effect of HFD on gene expression in adipose tissues.

In contrast with the activated thermogenic program in SC, the changes of thermogenic gene expression in BAT of *T-QKO* were relatively modest. It has been already reported that thermogenic capacity is antagonistically regulated in BAT and SC (Wu et al., 2014) (Kita et al., 2019). Therefore, this might be mainly caused by the impaired BAT activity by compensatory inhibition on BAT due to increased thermogenic program in SC. Furthermore, it might be easy to detect the differences of certain gene expression levels if mice could be kept in thermoneutral condition for at least 2 weeks before cold exposure.

The crosstalk between immune cells and adipocytes has attracted much attention as a place of activation of brown adipocytes and generation of beige adipocytes. Type 2 cytokines are produced by various immune cells, including eosinophils, ILC2s, Th2 cells, and Tfh cells (de Kouchkovsky et al., 2017). The present study indicates that IL-4 and IL-13 are secreted by Th2 and/or Tfh cells because expressions of the *Gata3* gene (a master gene of Th2 cells) and *Bcl6* and *Cxcr5* (markers of Tfh cells) are significantly increased in adipose tissues and sorted CD4^+^ T cells from *T-QKO*. In contrast, no significant change of *Siglecf* expression can exclude the possibility of eosinophil involvement. Furthermore, although Gata3 is also important for ILC2s, expression levels of *Rora* and *Il33r* (other marker genes of ILC2s) (Spits et al., 2013) in *T-QKO* mice were similar or reduced compared with control mice. Therefore, it is also difficult to conclude that ILC2s are a source of type 2 cytokines.

Interestingly, although the number of Th2 cells in SC and BAT from *T-QKO* were not increased at room temperature, cold exposure for 12 h increased the number of Th2 cells in SC from *T-QKO*. In contrast, the number of Th2 cells in peripheral blood and spleen from *T-QKO* were significantly reduced compared with control mice. These data suppose the possibility that the chemokine-chemokine receptor system between adipose tissues and Th2 cells might be activated in a tissue-specific manner under cold environment. Indeed, Th2 cells of *T-QKO* have much amount of *Ccr4* and increase sensitivity to chemokines, including Ccl22. Moreover, cold exposure increases *Ccl22* expression in SC. Therefore, Th2 cells of *T-QKO* are prone to accumulate in SC. Ccl22 is also called as macrophage-derived chemokine (MDC) and expressed in dendritic cells, natural killer cells, and monocytes (Godiska et al., 1997) and is the high-affinity ligand of Ccr4 (Imai et al., 1998). The increased expression of *Ccl22* in SC from *T-QKO* might result from the different environment in adipose tissues, including each cell population of macrophages and dendritic cells, due to lean phenotype of *T-QKO*. Our results suggest that Ccr4 and Ccl22 are important for the recognition of adipose tissues by circulating Th2 cells and for directing Th2 cells to cold-induced thermogenesis in adipose tissues. Furthermore, *Gata3*, *Il4*, and *Il13* expression were increased in adipose tissues of *T-QKO* mice. At this time, we think that the inhibition of *Gata3* expression by Foxo is a critical point of regulation for energy metabolism because Gata3 is a Th2 lineage-determining factor and promotes Th2 cytokine (Ouyang et al., 2000) and *Ccr4* expression (Yoshie and Matsushima, 2015). Because HFD activates Foxo1 in CD4^+^ T cells in adipose tissues, activation of Foxo1 in CD4^+^ T cells under HFD may suppress gene expression of *Gata3* and *Ccr4*, resulting in a decrease of homing of Th2 cells into adipose tissues, inhibition of brown and beige adipocytes, and decreased energy expenditure, finally leading to a vicious cycle of obesity. However, for the definite conclusion regarding the adipose tissue-specific homing of Th2 cells being required for the thermogenic program regulation, further investigations should be performed.

Foxo family members include the known cell-type-specific Foxo target genes that profoundly affect T cell survival, homing, proliferation, and differentiation (Hedrick et al., 2012). Foxo1 and Foxo3 in CD4^+^ T cells are the most important among Foxo family members because the *T-DKO* mice are lethal from eight weeks of age due to immunological disturbance, leading to lymphoma, enteritis, and digestive malabsorption (Ouyang et al., 2010). Therefore, it is difficult to use *T-DKO* mice to analyze metabolic phenotypes, including body weight, glucose tolerance, and insulin sensitivity. *T-DKO* mice exhibit weight loss due to intestinal inflammation (Ouyang et al., 2010). However, *T-QKO* mice exhibited no histological findings of enteritis, no significant signs of malabsorption, and no signs of lymphoma. Interestingly, in the present study, *T-Foxo1KO*, *T-Foxo3KO*, and *T-rQKO* mice have no apparent phenotypes under either an NCD or an HFD. These suggest that, in CD4^+^ T cells, the actions of Foxo1 and Foxo3 in CD4^+^ T cells are redundant to each other, but the role of Foxo1 is more physiologically important than that of Foxo3. Indeed, it has been suggested that Foxo1 seems to have a role distinct from that of Foxo3, although the underlying basis for their intricately controlled and opposing functions is presently unknown (Hedrick et al., 2012).

It has already been reported that Foxo1 regulates Tfh cell differentiation through inducible T cell co-stimulator (ICOS) signaling. Specifically, Foxo1 negatively regulates *Bcl6* expression, which is consistent with our results (Stone et al., 2015). However, whether *Gata3* expression is regulated by Foxo family members has not been reported. Interestingly, the *Gata3* promoter region has two consensus Foxo-binding sequences, TGTTTA (−1892 ~ −1887) and GTAAACA (−3873 ~ −3867) (Furuyama et al., 2000), at around 2 and 4 kb upstream of the transcription start site, respectively. These sequences are completely conserved in human, mouse, and rat *GATA3* promoter regions (Figures S9A and S9B). Furthermore, one of them, TGTTTA, is also completely conserved among chicken and zebrafish (Figure S8B). These findings support that Gata3 may also be a target of Foxo family members, and its expression is negatively regulated by Foxo although further investigation is needed. In contrast, the evolutional appearance of Ccr4 is relatively recent (Nomiyama et al., 2011), and there are no significantly conserved Foxo-binding sequences as far as examined. Therefore, Foxo suppresses *Gata3* expression, leading to reduced *Ccr4* expression.

Insulin receptor expression is not detectable on murine T cells in their resting state but rises in activated T cells, and insulin receptor signaling is an important node integrating pathway to drive optimal T cell effector function in health and disease (Tsai et al., 2018). Interestingly, diminished insulin-stimulated AKT signaling has been documented in the total lymphocytes of obese individuals (Viardot et al., 2007), and reduced expression of insulin receptor and downstream signaling molecules have also been reported in patients with type 2 diabetes (Stentz and Kitabchi, 2007). Therefore, an HFD might cause insulin resistance and activate Foxo family members in T cells. Furthermore, because Foxo family members are activated by oxidative stress, which is increased in adipose tissues under HFD (Kawano et al., 2012), we cannot exclude the possibility that HFD may activate Foxo1 through oxidative stress.

In humans, high levels of brown and beige adipocyte activity correlate with leanness, suggesting an important natural role for brown and beige adipocytes in human metabolism (Cypess et al., 2009, Saito et al., 2009, van Marken Lichtenbelt et al., 2009). Therefore, for an effective strategy to treat metabolic diseases, it is important to understand the molecular mechanism underlying the functional regulation of the amount and/or activity of brown and beige adipocytes. Data presented here demonstrate that Foxo1 and Foxo3 in CD4^+^ T cells likely act as a metabolic regulator that can suppress beiging with the potential capacity to accelerate obesity-induced insulin resistance. Our studies indicate a direct link between immune regulation through Foxo in T cells and energy homeostasis. Therefore, both Foxo1 and Foxo3 in CD4^+^ T cells should be molecular targets for the prevention and treatment of obesity. *T-QKO* in the present study is not a model of complete loss-of-*Foxo*. Alternatively, it is suggested that gene-dosage of *Foxo* in T cells regulates the recruitment of Th2 cells into adipose tissues, regulates the thermogenic program, and might determine the predisposition to obesity.

### Limitations of the Study

In the present study, we demonstrated that cold exposure induced Ccl22 expression, leading to increased accumulation of Th2 cells, in which loss-of-Foxo induced Ccr4 expression, in SC of *T-QKO*. However, further analyses, including gain- or loss-of-function studies of Ccl22, are required to elucidate the mechanism of cold-exposure-induced recruitment of Th2 cells through Ccr4/Ccl22 axis.

## Methods

All methods can be found in the accompanying Transparent Methods supplemental file.

## Supplemental Information

Document S1. Transparent Methods and Figures S1–S9
