# Supplementary material for: Foxo in T Cells Regulates Thermogenic Program through Ccr4/Ccl22 Axis
Source: iScience. 2019 Nov 7;22:81–96. doi: 10.1016/j.isci.2019.11.006 (PMC6880116; doi:10.1016/j.isci.2019.11.006)
Supplement: Document S1. Transparent Methods and Figures S1–S9 [file mmc1.pdf]

ISCI, Volume *22*

Supplemental Information

Foxo in T Cells Regulates Thermogenic Program through Ccr4/Ccl22 Axis

Tetsuhiro Kikuchi, Jun Nakae, Yoshinaga Kawano, Nobuyuki Watanabe, Masafumi Onodera, and Hiroshi Itoh

Supplemental Information

Foxo in T cells Regulates Thermogenic Program through Type 2 Immune Signaling

Tetsuhiro Kikuchi, Jun Nakae, Yoshinaga Kawano, Nobuyuki Watanabe, Masafumi Onodera, Hiroshi Itoh

Transparent Methods

Antibodies

We purchased anti-FOXO1 (L27) polyclonal antibody from Cell Signaling Technology, anti-FOXO1A antibody (ab12161) from Abcam^{R}, anti-FOXO3A antibody (ab12162), and anti-CD4 antibody (100505) from BioLegend. For histological analysis, we used anti-CD68 (Dako Denmark A/S) and anti-UCP1 antibodies (Santa Cruz Biotechnology Inc).

Available Mice

Conditional *Foxo1*$^{flox/flox}$, *Foxo3*$^{flox/flox}$ (Paik et al., 2007) and *CD4*$^{+}$*-Cre* (Sawada et al., 1994) mice have been previously described elsewhere.

Animal Studies, Analytical Procedures, and Intraperitoneal Glucose and Insulin Tolerance Tests

For the following experiments, we used only male mice because they are more susceptible to insulin resistance and diabetes. Mice were housed in a barrier animal facility at 22–24°C with a 12-h light/dark cycle. For HFD (HFD-60; Oriental Yeast Co. Ltd.) studies, we started the HFD at 4 weeks of age. All of the HFD mice were compared to age-matched mice fed an NCD. The composition of HFD used in this study is described previously (Kawano et al., 2016). The IPGTT, ITT, and insulin measurements were performed at 20 to 22 weeks of age as described previously (Kawano et al., 2016). All assays were performed in duplicate, and each value represents the mean of two independent determinations. The rectal temperature of mice was measured at 16 weeks of age using Thermal SensorR (Shibaura Electronics Co., Ltd). The studies of gene expression at room temperature, measurements of tissue weights, and of adipocyte size were performed at 20-24 weeks of age. Food intake was monitored by weighing the chow every 24 h for 2 weeks using male 12-week-old mice housed individually. All experimental protocols using mice were approved by the animal ethics committees of the Keio University School of Medicine.

Measurement of Oxygen Consumption.

Mice aged 10 to 12 weeks under HFD were monitored individually in a metabolic cage (ARCO-2000; ARCO SYSTEM Inc., Kashiwa, Japan.) with free access to HFD and drinking

water for 72 h. Each cage was monitored for oxygen consumption at 5-min intervals for 72 h, with the first day allowing the mice to acclimate to the cage environment. Total oxygen consumption was calculated as accumulated oxygen uptake for each mouse. We measured oxygen consumption of 8 mice in each genotype. Representative graphs were drawn from mean $\pm$ SEM values calculated from data obtained in each measurement.

Cold Exposure

For experiments at cold exposure, 16-week-old mice were placed in individual cages at 4°C for 6 h ~ 12 h with free access to food and drinking water. For FACS analysis, mice were placed at 4°C for 12 h.

Immunohistochemistry, Immunofluorescence and Histological Analysis

For histological analysis, we removed the WAT, small intestine, and colon from 20- to 24-week-old mice, fixed the specimens in 4% paraformaldehyde and embedded them in paraffin. We mounted consecutive 10μm sections on slides. After rehydration and permeabilization, we stained the specimens with hematoxylin and eosin. Immunohistochemistry was performed as described previously (Kawano et al., 2012) using anti-CD68 and anti-UCP1antibody. After a wash with phosphate-buffered saline, the sections were sequentially incubated with secondary antibody and visualized using the Liquid DAB Substrate Chromogen System (DakoCytomation). The size and number of adipocytes in WAT were determined using a fluorescence microscope (BZ-8000, 9000, KEYENCE) by manually tracing at least more than 1000 adipocytes for each genotype (n=8-10). Measurement of number of CLSs was performed at 20 to 24 weeks of age as described previously (Fujisaka et al., 2009). Measurement of crypt depth and the number of goblet cells in colon and small intestine were performed using a microscope by manually tracing at least 100 crypts for each genotype (n=4). For immunofluorescence analysis, epididymal fat was dissected and immersed in 4% paraformaldehyde at 4°C overnight and soaked in 30% sucrose overnight. For double-staining with CD4 and Foxo1, the secondary antibody for an anti-mouse CD4 was Alexa FluorR 488 goat anti-mouse IgG, and the anti-Foxo1 antibody was Alexa FluorR 594 chicken anti-rabbit IgG (Molecular Probes, Eugene, OR). For the quantification of CD4 and Foxo1 in $CD4^+$ cells, tissues were processed as described above

for the double staining procedures. Pictures were taken of two mice for the same HFD duration. T cells double-positive for CD4 and Foxo1 (cytoplasmic, nuclear, or both) immunoreactivity were counted and marked digitally to prevent multiple counts with Adobe Photoshop CS4 EXTENDEDR and ImageJ software (NIH; Bethesda, MD). Cell counts were performed in three mice for the same HFD duration. At least 300 cells were counted in each mouse.

RNA Isolation and Real-time PCR

Isolation of total RNA was performed using the SV Total RNA Isolation System (Promega) according to the manufacturer’s protocol. We performed reverse transcription using the PrimeScriptTM RT Reagent Kit, and real-time PCR using the SYBR GREEN detection protocol by STRATAGENE (An Agilent Technologies Division, Germany). All primer sequences are available upon request.

Flow Cytometry Analysis

Firstly, spleen was removed and cut into small pieces with scissors and then filtered through 40μm nylon mesh. The cells were collected in a new 50 ml tube, and the supernatants were centrifuged at 1500 rpm for 5min at 4℃ and washed twice with PBS. After hemolytic incubation with lysing solution (BD), the cells were washed twice again and analyzed by FACS immediately. Cells were incubated in Pharm Lyse (BD Biosciences) with Fc block (1:100) for 15 min at 4°C. Cells were stained with primary antibodies or the matching control isotypes for 30 min at 4°C in dark, and then washed twice. They were re-suspended in PBS and stained with 7-Aminoactinomycin (7AAD). The cells were analyzed using FACSAriaⅢ (BD). The data were analyzed with FlowJo. The following antibodies were used: anti-CD3 (100204) and anti-CD4 antibodies (100430) from BioLegend, anti-CD8 (563152), anti-CXCR3 (562266), anti-CXCR5 (560617), anti-CCR6 (564736), and anti-PD-1 (744544) from BD Biosciences. Samples for RNA analysis were collected directly in the SV Total RNA Isolation System (Promega) reagent.

Magnetic Activated Cell Sorting

$CD4^+$ T cells were isolated from adipose tissues and incubated with CD4 magnetic beads

(Miltenyi Biotec) for positive selection. The $CD4^+$ cells were cultured with (2×10^6 cells/ml) in HANKs' Balanced Salt solution supplemented with 1.5% FCS and 1% penicillin/streptomycin.

Triglyceride Measurements in Liver and Stool

Liver and stool homogenates were extracted, and triglyceride content was determined as described (Murakami et al., 1998) with an extract solution (CHCl3:CH3OH = 2:1) using Triglyceride E-test WAKO (FUJIFILM-WAKO).

Measurement of IL1β

Serum levels of IL1βwere measured using the mouse ELISA kits (mouse ELISA kit Quantikine, R&D SYSTEM for IL1β).

Gut Microbiota Analysis

DNA sample for assessment of microbial community was extracted from lyophilizated cecal content using QIAamp DNA Stool Mini Kit (Qiagen). Real time PCR was performed using with LightCycler 480 System II (Roche) and SYBR Freen I Master (Roche). Analysis Object of bacterial phylum were Firmicutes and Bacteroides.

Statistical Analysis

We calculated descriptive statistics using one-way or two-way ANOVA with Fisher's test. All data are expressed as mean + standard error (SEM). Significance was set at $p<0.05$.

SUPPLEMENTAL REFERENCES

Fujisaka, S., Usui, I., Bukhari, A., Ikutani, M., Oya, T., Kanatani, Y., Tsuneyama, K., Nagai, Y., Takatsu, K., Urakaze, M., *et al.* (2009). Regulatory mechanisms for adipose tissue M1 and M2 macrophages in diet-induced obese mice. Diabetes *58*, 2574-2582.

Kawano, Y., Nakae, J., Watanabe, N., Fujisaka, S., Iskandar, K., Sekioka, R., Hayashi, Y., Tobe, K., Kasuga, M., Noda, T., *et al.* (2012). Loss of Pdk1-Foxo1 signaling in myeloid cells predisposes to adipose tissue inflammation and insulin resistance. Diabetes *61*, 1935-1948.

Kawano, Y., Nakae, J., Watanabe, N., Kikuchi, T., Tateya, S., Tamori, Y., Kaneko, M., Abe, T., Onodera, M., and Itoh, H. (2016). Colonic Pro-inflammatory Macrophages Cause Insulin Resistance in an

Intestinal Ccl2/Ccr2-Dependent Manner. Cell Metab *24*, 295-310.

Murakami, K., Tobe, K., Ide, T., Mochizuki, T., Ohashi, M., Akanuma, Y., Yazaki, Y., and Kadowaki, T. (1998). A novel insulin sensitizer acts as a coligand for peroxisome proliferator-activated receptor-alpha (PPAR-alpha) and PPAR-gamma: effect of PPAR-alpha activation on abnormal lipid metabolism in liver of Zucker fatty rats. Diabetes *47*, 1841-1847.

Paik, J.H., Kollipara, R., Chu, G., Ji, H., Xiao, Y., Ding, Z., Miao, L., Tothova, Z., Horner, J.W., Carrasco, D.R.*, et al.* (2007). FoxOs Are Lineage-Restricted Redundant Tumor Suppressors and Regulate Endothelial Cell Homeostasis. Cell *128*, 309-323.

Sawada, S., Scarborough, J.D., Killeen, N., and Littman, D.R. (1994). A lineage-specific transcriptional silencer regulates CD4 gene expression during T lymphocyte development. Cell *77*, 917-929.

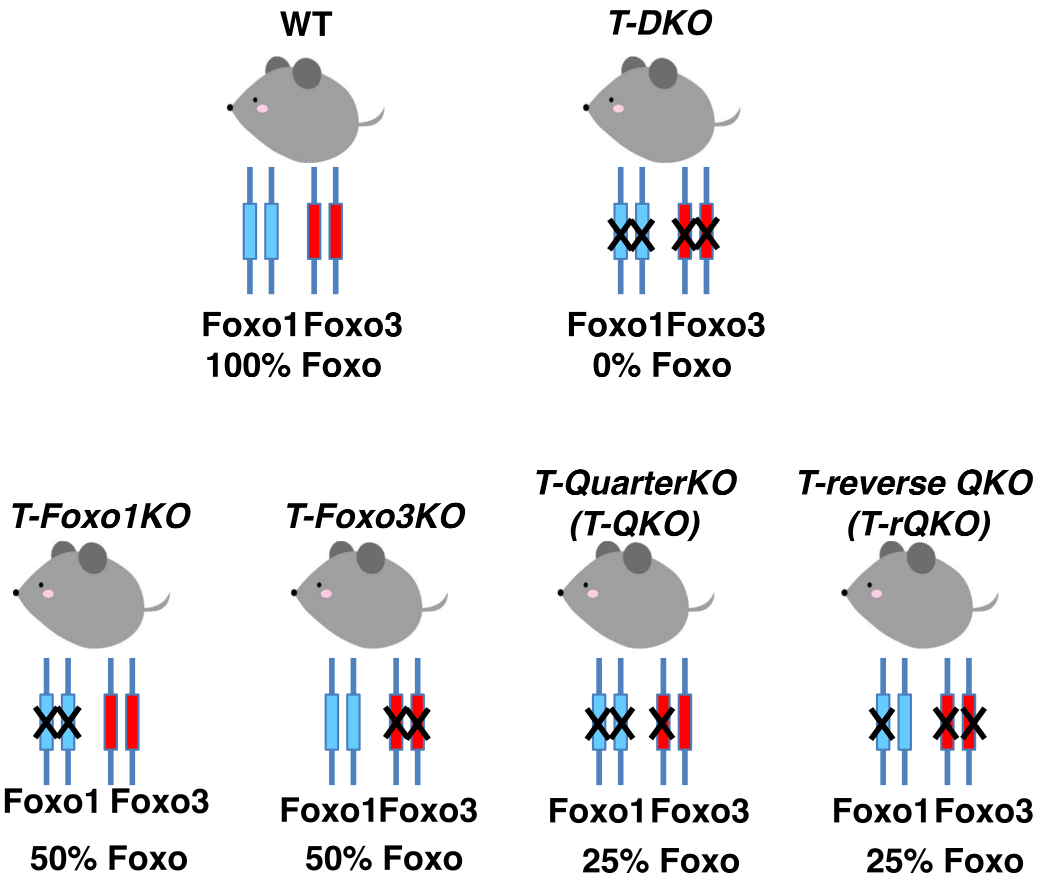

WT
T-DKO
Foxo1 Foxo3
100% Foxo
Foxo1 Foxo3
0% Foxo
T-Foxo1KO
T-Foxo3KO
T-QuarterKO
(T-QKO)
T-reverse QKO
(T-rQKO)
Foxo1 Foxo3
50% Foxo
Foxo1 Foxo3
50% Foxo
Foxo1 Foxo3
25% Foxo
Foxo1 Foxo3
25% Foxo

Figure S1

Figure S1. Related to Figure 2 and Figure 3. Schema of CD4$^+$ T Cell-specific *Foxo* Knockout Mice.

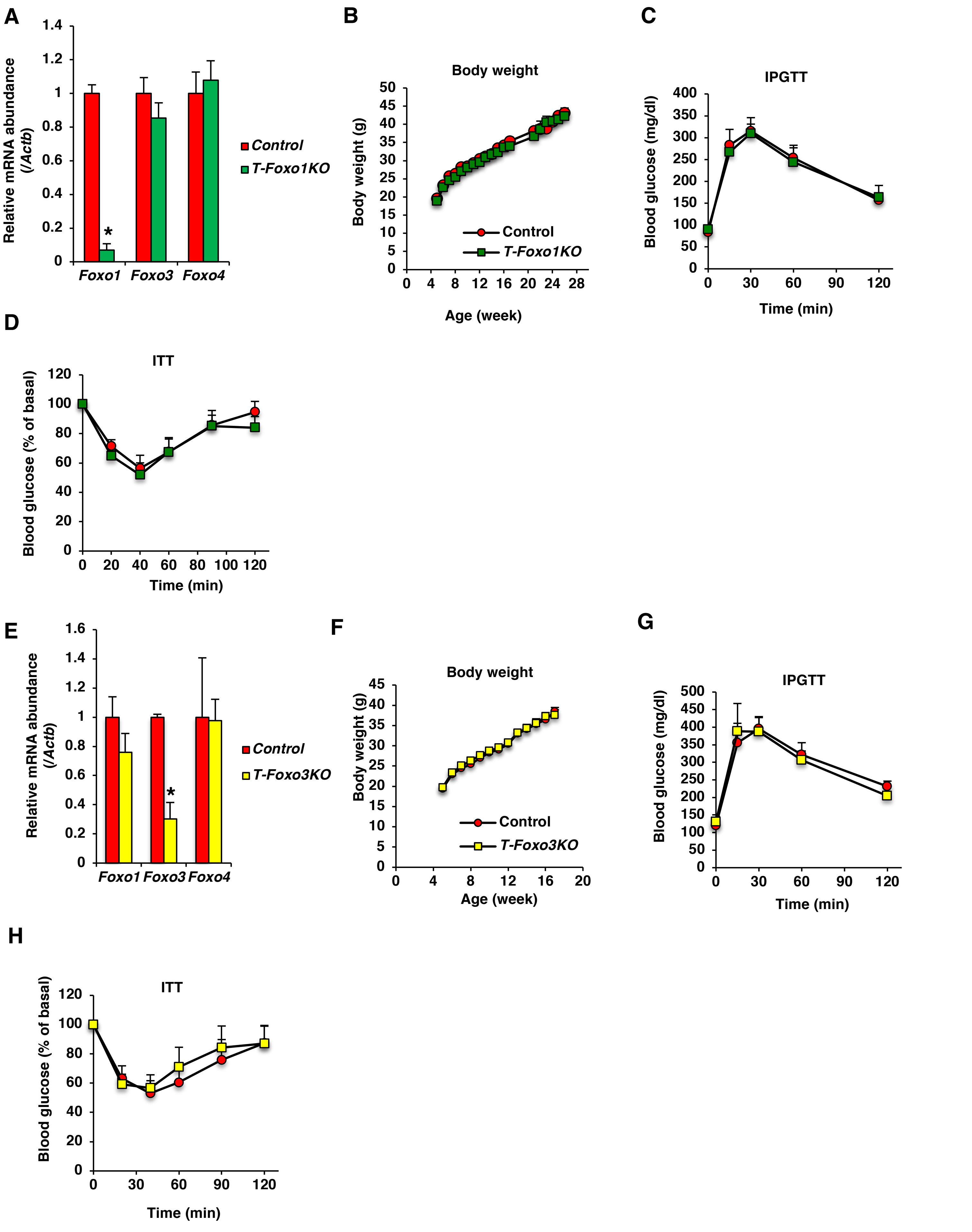
A
Relative mRNA abundance (/Actb)
Control
T-Foxo1KO
Foxo1 Foxo3 Foxo4
B
Body weight
Body weight (g)
Control
T-Foxo1KO
Age (week)
C
IPGTT
Blood glucose (mg/dl)
Time (min)
D
ITT
Blood glucose (% of basal)
Time (min)
E
Relative mRNA abundance (/Actb)
Control
T-Foxo3KO
Foxo1 Foxo3 Foxo4
F
Body weight
Body weight (g)
Control
T-Foxo3KO
Age (week)
G
IPGTT
Blood glucose (mg/dl)
Time (min)
H
ITT
Blood glucose (% of basal)
Time (min)

Figure S2

Figure S2. Related to Figure 2, *Foxo1* or *Foxo3* in $CD4^+$ T Cells is Dispensable for Glucose and Energy Metabolism.

(A) Expression of *Foxo1, Foxo3,* and *Foxo4* in $CD4^+$ T cells sorted from spleen of control and *T-Foxo1KO* (n=4). Data are normalized to b-actin expression. Data are means $\pm$ SEM. *P<0.05 by one-way ANOVA.

(B) Body weight of control (the red circle) and *T-Foxo1KO* mice (the green square) fed with HFD (n=13). Data are means $\pm$ SEM.

(C) IPGTT of control and *T-Foxo1KO* mice fed with HFD for 16 weeks (n=7). Data are means $\pm$ SEM.

(D) ITT of control and *T-Foxo1KO* mice fed with HFD for 16 weeks (n=7). Data are means $\pm$ SEM.

(E) Expression of *Foxo1, Foxo3,* and *Foxo4* in $CD4^+$ T cells sorted from spleen of control and *T-Foxo3KO* (n=4). Data are normalized to b-actin expression. Data are means $\pm$ SEM. *P<0.05 by one-way ANOVA.

(F) Body weight of control (the red circle) and *T-Foxo3KO* mice (the yellow square) fed with HFD (n=13). Data are means $\pm$ SEM.

(G) IPGTT of control and *T-Foxo3KO* mice fed with HFD for 16 weeks (n=4). Data are means $\pm$ SEM.

(H) ITT of control and *T-Foxo3KO* mice fed with HFD for 16 weeks (n=4). Data are means $\pm$ SEM.

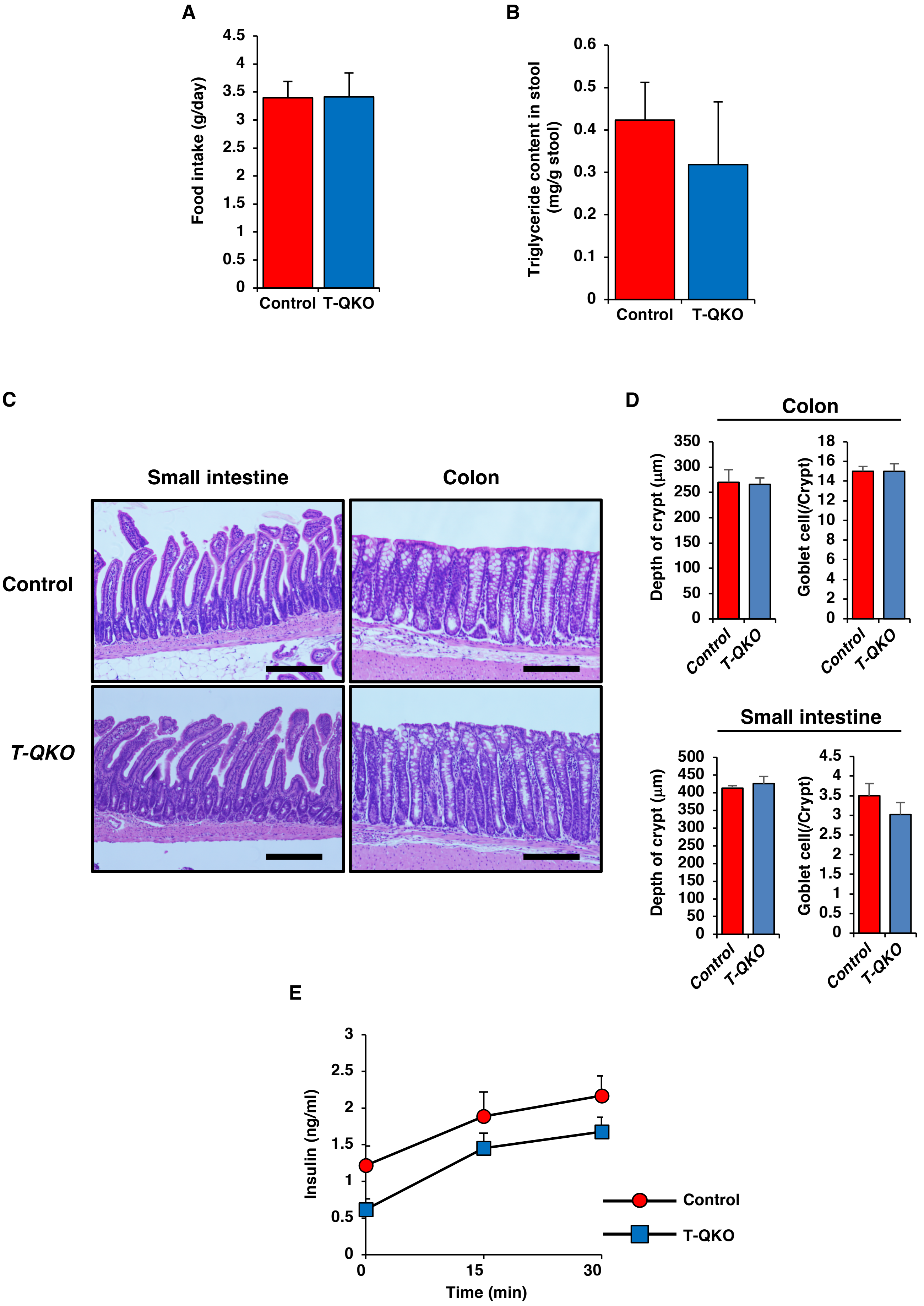

A
Food intake (g/day)
4.5
4
3.5
3
2.5
2
1.5
1
0.5
0
Control
T-QKO
B
Triglyceride content in stool (mg/g stool)
0.6
0.5
0.4
0.3
0.2
0.1
0
Control
T-QKO
C
Small intestine
Colon
Control
T-QKO
D
Colon
Depth of crypt (μm)
Goblet cell(/Crypt)
Control
T-QKO
Small intestine
Depth of crypt (μm)
Goblet cell(/Crypt)
Control
T-QKO
E
Insulin (ng/ml)
Time (min)
0
15
30
Control
T-QKO

Figure S3

Figure S3. Related to Figure 2. Metabolic Phenotype of *T-QKO* under HFD.

(A) Food intake (g/day) of control and *T-QKO* fed with a 8-week HFD (n=6). Data represent mean ± SEM of food intake for 4 days.

(B) Triglyceride content in stool of control and *T-QKO* fed with a 6-week HFD (n=4). Data represent mean ± SEM.

(C) Representative hematoxylin eosin-staining images of small intestine and colon from control and *T-QKO* fed with HFD for 10 weeks (scale bar, 100mm).

(D) Quantification of depth of crypt and number of goblet cells in crypt in colon (upper panel) and small intestine (bottom panel). Data are means ± SEM of 5 mice in each genotype.

(E) Insulin secretion of *T-QKO* during IPGTT. Data are means ± SEM of 5-8 mice in each genotype.

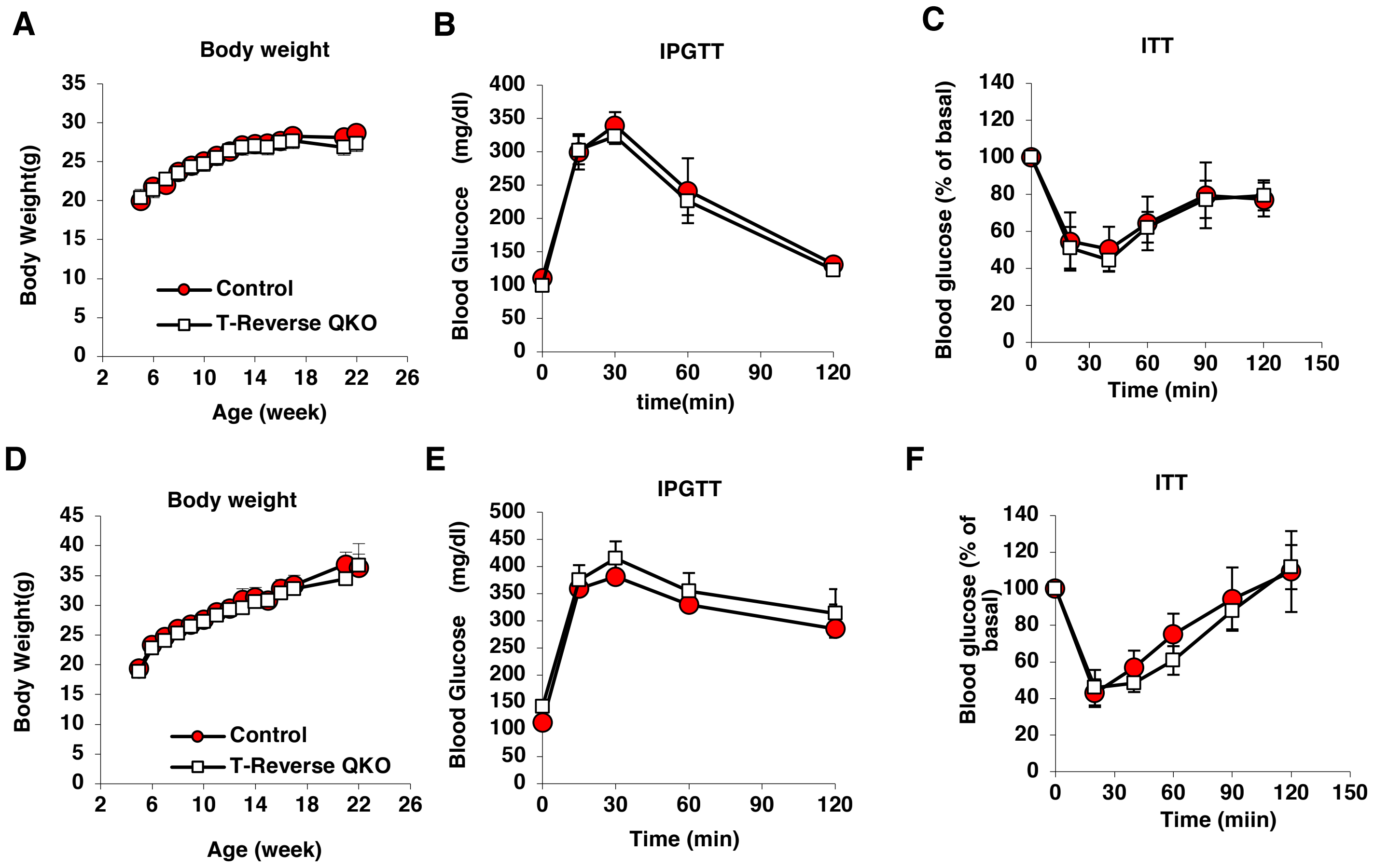
A
Body weight
Body Weight(g)
Age (week)
Control
T-Reverse QKO
B
IPGTT
Blood Glucose (mg/dl)
time(min)
C
ITT
Blood glucose (% of basal)
Time (min)
D
Body weight
Body Weight(g)
Age (week)
Control
T-Reverse QKO
E
IPGTT
Blood Glucose (mg/dl)
Time (min)
F
ITT
Blood glucose (% of basal)
Time (miin)

Figure S4

Figure S4. Related to Figure 2. Glucose Metabolism of *T-rQKO* under NCD and HFD.

(A)(D) Body weight of control (red circle) and *T-rQKO* mice (white square) fed with NCD (A) and HFD (D) (n=13). Data are means ± SEM.

(B)(E) IPGTT of control and *T-rQKO* mice fed with NCD at the age of 21 weeks (n=5) (B) and 16-week HFD at the age of 21 weeks (n=7) (E). Data are means ± SEM.

(C)(F) ITT of control and *T-rQKO* mice fed with NCD at the age of 21 weeks (n=4) (C) and 16-week HFD at the age of 21 weeks (n=11) (F). Data are means ± SEM.

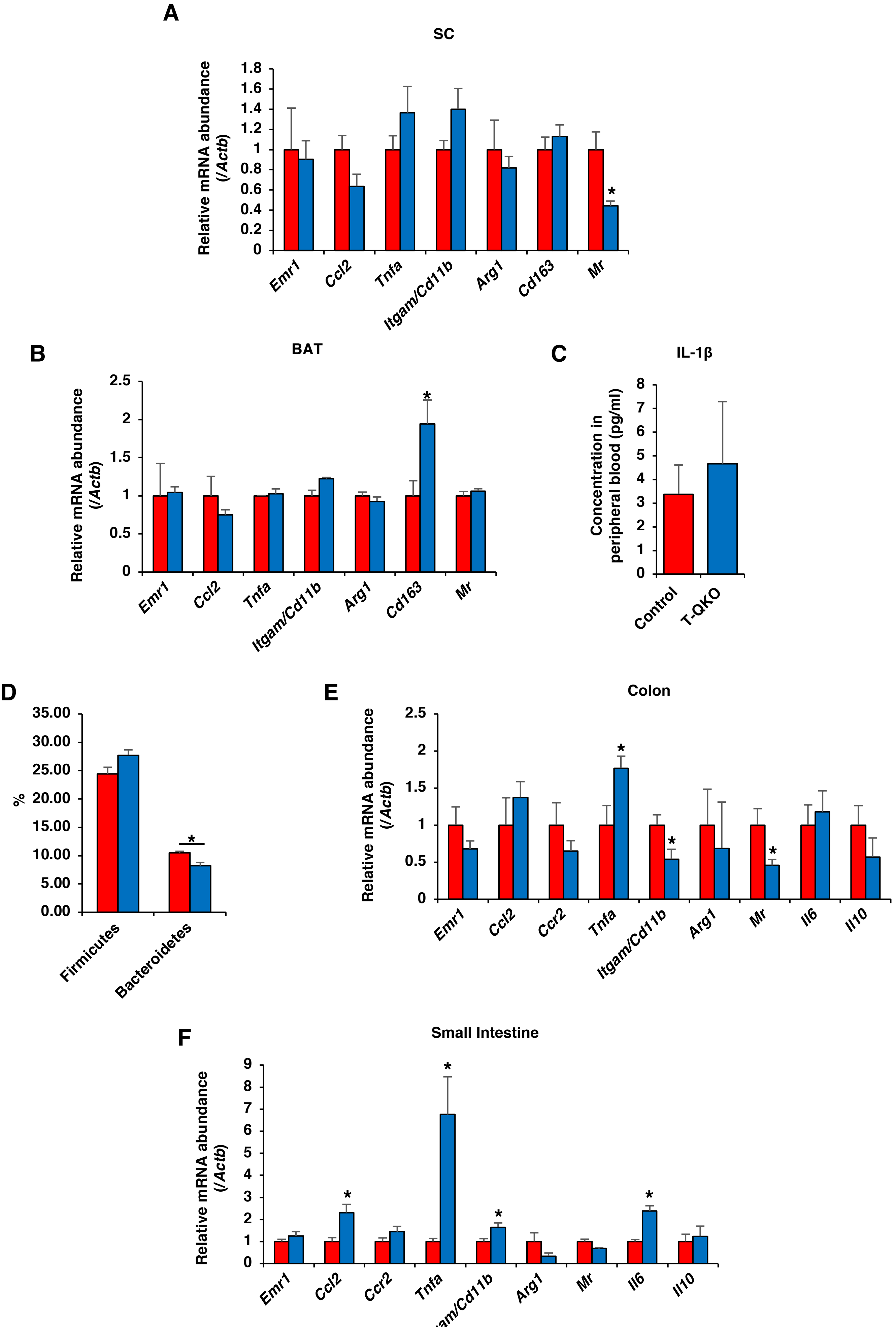
A
SC
Relative mRNA abundance (/Actb)
Emr1
Ccl2
Tnfa
Itgam/Cd11b
Arg1
Cd163
Mr
B
BAT
C
IL-1β
Concentration in peripheral blood (pg/ml)
Control
T-QKO
D
%
Firmicutes
Bacteroidetes
E
Colon
Ccr2
Il6
Il10
F
Small Intestine

Figure S5

Figure S5. Related to Figure 3. Normalized Gene Expression Levels of Immune Cell-related Genes in Several Tissues.

(A)(B)(E)(F) Normalized gene expression of immune cell-related genes in SC (A), BAT(B), colon (E), and small intestine (F) of control and *T-QKO* fed with a 20-week HFD at room temperature (n=6). Data are the ratio of control in each gene and means $\pm$ SEM. *p<0.05 by one-way ANOVA.

(C) The concentration of IL-1β in peripheral blood of control and *T-QKO* fed with HFD for 20 weeks (n=6).

(D) The percentage of Firmicutes and Bacteroides in the cecum flora from control (red bar) and *T-QKO* (blue bar) fed with HFD for 20 weeks. *p<0.05 by one-way ANOVA.

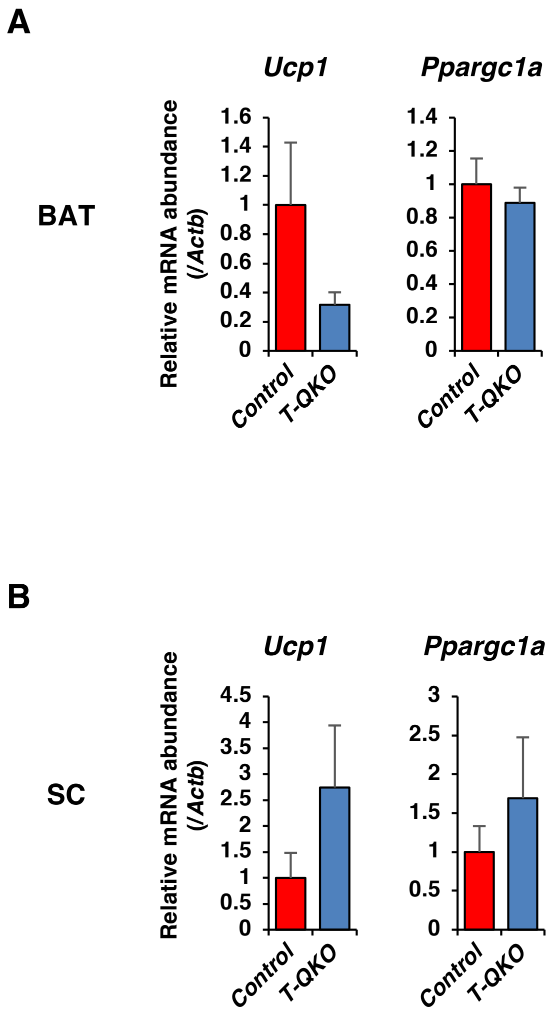
A
Ucp1
Ppargc1a
BAT
Relative mRNA abundance (/Actb)
1.6
1.4
1.2
1
0.8
0.6
0.4
0.2
0
Control
T-QKO
B
SC
4.5
4
3.5
3
2.5
2
1.5
0.5

Figure S6

Figure S6. Related to Figure 4. Gene Expression of *Ucp1* and *Ppargc1a* in BAT (A) and SC (B) in Control and *T-QKO* Fed with NCD at Room Temperature.

(A)(B) Normalized gene expression of *Ucp1* and *Ppargc1a* in BAT (A) and SC (B) of 21-week-old control and *T-QKO* fed with NCD at room temperature (n=5). Data are the ratio of control in each gene and means ± SEM.

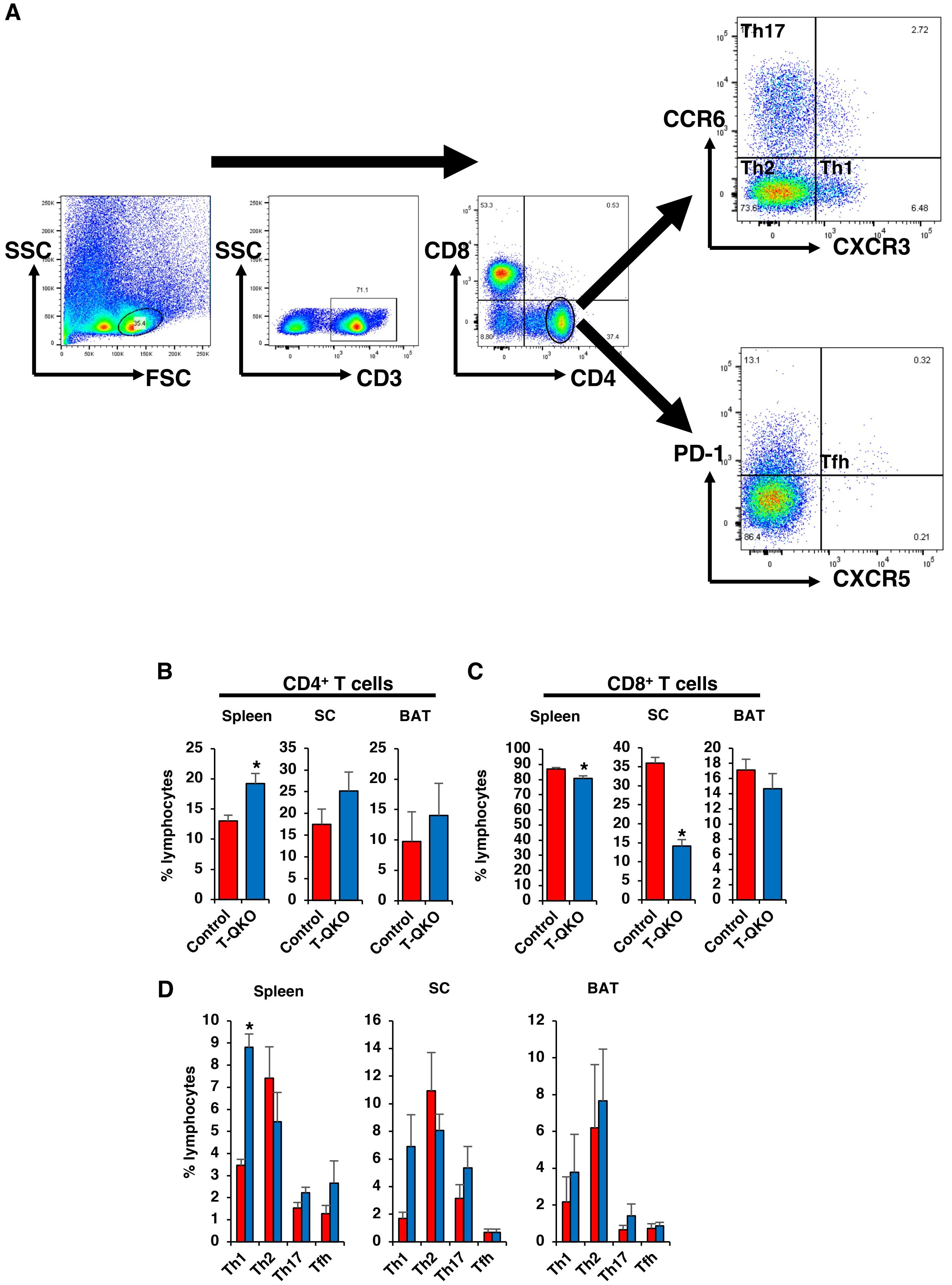
A
SSC
FSC
SSC
CD3
CD8
CD4
CCR6
CXCR3
Th17
Th2
Th1
PD-1
CXCR5
Tfh
B
CD4+ T cells
Spleen
SC
BAT
% lymphocytes
Control
T-QKO
C
CD8+ T cells
D
Th1
Th2
Th17
Tfh

Figure S7

Figure S7. Related to Figure 5. *Foxo* Loss in $CD4^+$ T Cells Does Not Affect the Numbers of Th2 Cells in SC and BAT at Room Temperature.

(A) Representative Gating Strategy for T Helper Cells Analysis.

(B)(C) FACS analysis of $CD4^+$ (B) and $CD8^+$ (C) T cells in spleen, SC, and BAT from control and *T-QKO* fed with a 20-week HFD at room temperature (n=4). Data are the percentage of lymphocytes and means $\pm$ SEM. *p<0.05 by one-way ANOVA.

(D) FACS analysis of Th1, Th2, Th17, and Tfh cells in spleen, SC and BAT from control and *T-QKO* fed with a 20-week HFD at room temperature (n=4). Data are the percentage of lymphocytes and means $\pm$ SEM. *p<0.05 by one-way ANOVA.

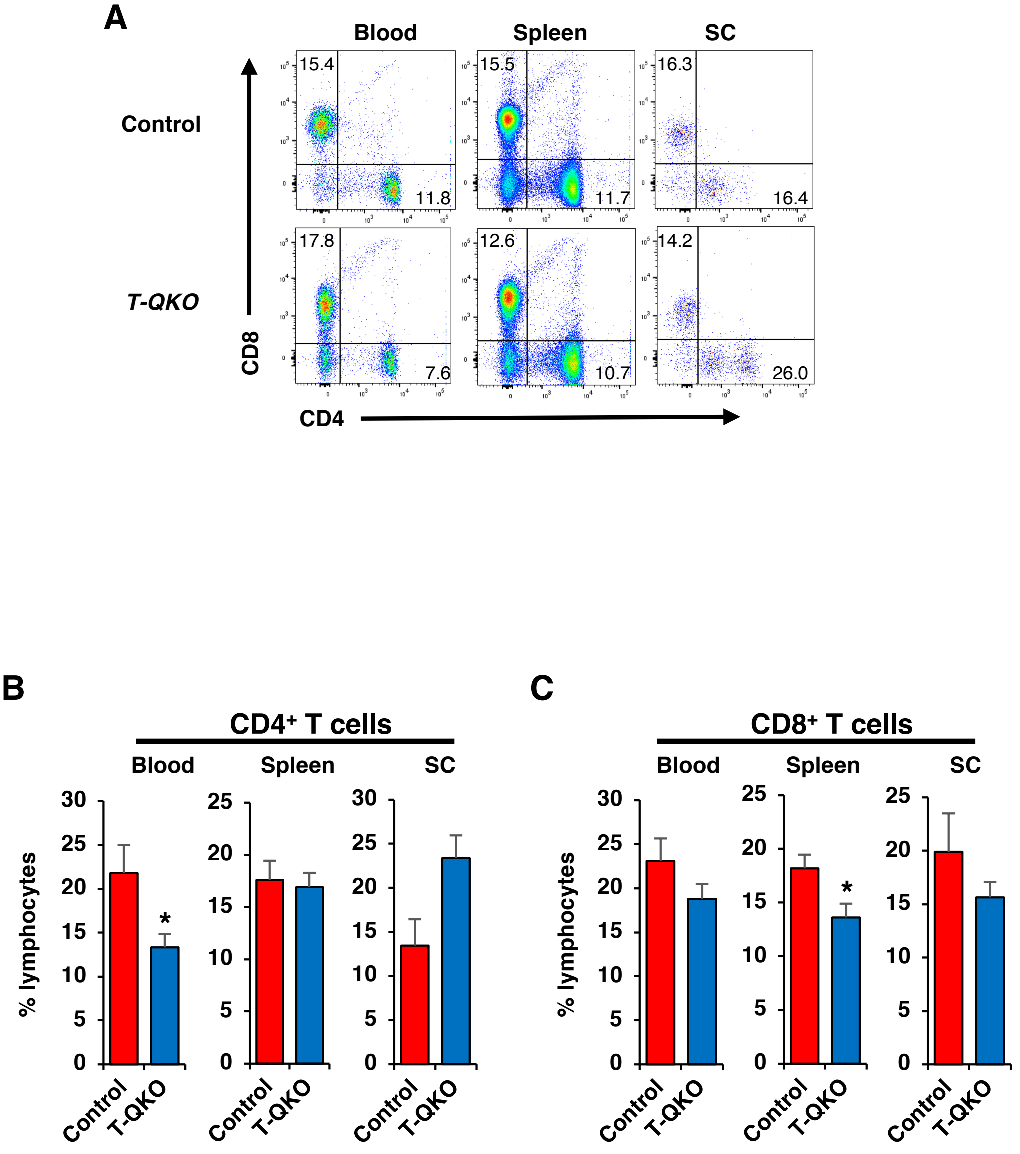
A
Blood
Spleen
SC
Control
T-QKO
CD8
CD4
15.4
11.8
15.5
11.7
16.3
16.4
17.8
7.6
12.6
10.7
14.2
26.0
B
CD4⁺ T cells
Blood
Spleen
SC
% lymphocytes
Control
T-QKO
*
C
CD8⁺ T cells
Blood
Spleen
SC
% lymphocytes
Control
T-QKO
*

Figure S8

Figure S8. Related to Figure 5. The Effects of *Foxo* Loss in CD4$^+$ T Cells on Cell Populations of CD4$^+$ and CD8$^+$ T Cells at Cold Exposure.

(A-C) FACS analysis of lymphocytes from peripheral blood (Blood), spleen, and subcutaneous adipose tissue (SC) of control and *T-QKO* fed with a 20-week HFD incubated at 4^oC for 12 hours (n=4).
(A) Surface CD4 and CD8 expression of lymphocytes. Bar graphs represent frequency of CD4$^+$ (B) and CD8$^+$ (C) T cells.
Data are the percentage of total lymphocytes and means $\pm$ SEM. *$p<0.05$ by one-way ANOVA.

A

Human	CAAAGTCTTTAA**GTAAACA**CGCTCAAATGAC	-3900~-3894
Mouse	CAAAGTCCCTAA**GTAAACA**CGCTCAAATGAC	-3873~-3867
Rat	CAAAGTCCTTAA**GTAAACA**CGCTCAAATGAC	-3881~-3875

B

Human	TTCGTTTCTCCT**TGTTTA**TGGAGAGGTTTC	-1995 -1990
Mouse	GTCTTCTCTGCT**TGTTTA**TGGAATGGTTTC	-1892~-1887
Rat	GTCTTCTCTGCT**TGTTTA**TGGAATGGTTTC	-1741~-1736
Chicken	TTAAAAGGAATT**TGTTTA**AAATGACGTTTT	-2088~-2083
Zebrafish 1	AGTGCACCATAG**TGTTTA**TTATTGATGTAT	-2694~-2689
Zebrafish 2	ATATTTCCTGCC**TGTTTA**CTGACGTCACGT	-1529~-1524
Zebrafish 3	TGATCAAACTTG**TGTTTA**GTCAGAAGGCAG	-263~-258

Figure S9

Figure S9. Related to Figure 5. Foxo Binding Sequences of *Gata3* Promoter Region are Conserved.
(A) Foxo binding sequence (GTAAACA) of *Gata3* promoter region of human, mouse, and rat.
(B) Foxo binding sequence (TGTTTA) of *Gata3* promoter region of human, mouse, rat, chicken, and zebrafish. The number indicates the location upstream from transcription start site.
